# Oligosaccharides With Defined Glycosidic Bonds Shape Gut Microbial Succession and Metabolism Via Bond‐Specific Microbial Responders

**DOI:** 10.1002/advs.77488

**Published:** 2026-09-01

**Authors:** Xiaoxuan Lu, Jiaqi Zou, Geng Han, Mengyao Zhao, Ting Luo, Xiangru Feng, Liangliang Zhu, Yijia Chen, Xiaoguo Ji, Jiayang Jin, Liming Zhao

**Affiliations:** ^1^ State Key Laboratory of Bioreactor Engineering East China University of Science and Technology Shanghai People's Republic of China; ^2^ Shanghai Frontiers Science Center of Optogenetic Techniques For Cell Metabolism School of Pharmacy East China University of Science and Technology Shanghai People's Republic of China; ^3^ Shanghai Collaborative Innovation Center For Biomanufacturing Technology (SCICBT) Shanghai People's Republic of China; ^4^ ECUST Medical‐Engineering Integration Innovation Center Shanghai People's Republic of China

**Keywords:** generalized lotka–volterra model, glycosidic bond, metaproteomics, microbial responder, short‐chain fatty acids, synthetic microbial community

## Abstract

Functional oligosaccharides are important prebiotic ingredients, but the structure‐function relationships and mechanisms by which defined glycosidic bonds shape microbial responses remain unclear. Five glucose disaccharides, trehalose (α‐1,1), maltose (α‐1,4), isomaltose (α‐1,6), cellobiose (β‐1,4), and gentiobiose (β‐1,6), were used as minimal oligosaccharide models to isolate glycosidic bond effects. Absolute time‐series profiling combined with Bayesian generalized Lotka–Volterra modeling identified bond‐specific microbial responders, operationally defined as taxa with statistically supported substrate‐associated growth advantages beyond endpoint dominance. α‐Linked disaccharides mainly recruited *Bifidobacterium pseudocatenulatum* and *Megamonas funiformis*, cellobiose enriched *Faecalibacterium prausnitzii*, and gentiobiose enriched *B. pseudocatenulatum*. Monoculture assays confirmed direct cognate disaccharide utilization. Metaproteomics revealed linkage‐matched modules: isomaltose responders upregulated GanO/ChvE and oligo‐1,6‐glucosidase; cellobiose responders expressed CebE/ChvE, ABC.MS.S, CelB, cellobiose phosphorylase, and β‐glucosidases; whereas the molecular evidence for gentiobiose was based mainly on ABC.MS.S and general β‐glucosidases. Metabolically, gentiobiose favored acetic acid accumulation, cellobiose yielded the highest butyric acid concentration, and isomaltose elevated trans‐4‐hydroxy‐L‐proline and 7,8‐dihydroneopterin associated with redox and immune‐related cofactor pathways. Guided by these ecological and molecular observations, microbial responder‐centered synthetic microbial communities utilized cognate disaccharides, recapitulated glycosidic bond‐specific ecological succession, showed greater net short‐chain fatty acid (SCFA) accumulation than matched complex communities under equal initial substrate input in vitro, and elevated fecal SCFAs in mice, with cellobiose increasing butyric acid by 2.1‐fold. These results support a mechanistically informed pathway linking glycosidic bond structure, microbial succession, and metabolic outputs, providing a basis for structure‑guided microbiome modulation.

## Graphical abstract

Oligosaccharides with defined glycosidic bonds drive divergent gut microbial succession and shape unique metabolic profiles. Absolute time‐series profiling with Bayesian generalized Lotka–Volterra modeling identifies bond‐specific microbial responders, while monoculture validation and metaproteomics reveal linkage‐matched transport and cleavage modules. These rules guide synthetic microbial community (SynCom) construction, enabling cognate substrate degradation and greater net SCFA accumulation than matched complex communities in vitro, together with modulation of gut barrier in pseudo‐germ‐free mice.

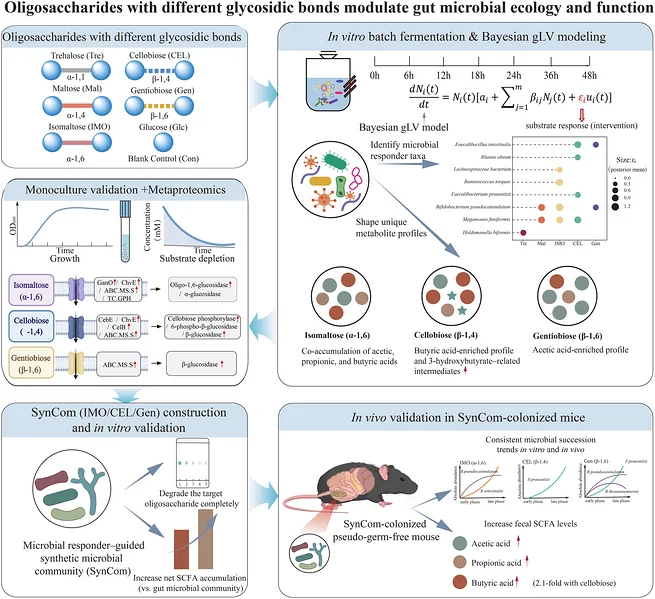

**Abbreviations**
ANOVA: Analysis of Variance
*B. obeum*
: *Blautia obeum*
*B. pseudocatenulatum*
: *Bifidobacterium pseudocatenulatum*
*B. thetaiotaomicron*
: *Bacteroides thetaiotaomicron*
CCS: Circular consensus sequencing
*F. intestinalis*
: *Faecalibacillus intestinalis*
*F. prausnitzii*
: *Faecalibacterium prausnitzii*
FC: Fold change
GC‐FID: Gas chromatography with flame ionization detection
gLV: Generalized Lotka–Volterra
H&E: Hematoxylin and Eosin
*H. biformis*
: *Holdemanella biformis*
*M. funiformis*
: *Megamonas funiformis*
MEBA: Multivariate empirical bayes analysis of time course
OPLS‐DA: Orthogonal partial least squares discriminant analysis
PBS: Phosphate buffered saline
PCA: Principal component analysis
PLS‐DA: Partial least squares discriminant analysis
*R. intestinalis*
: *Roseburia intestinalis*
*R. torques*
: *Ruminococcus torques*
RDA: Redundancy analysis
SCFA: Short‐chain fatty acid
SynComs: Synthetic microbial communities

## 1 Introduction

Functional oligosaccharides are important prebiotic substrates for gut microbiota modulation and are widely used in functional foods, nutritional products, and dietary interventions [1]. Beyond their application value, they provide chemically tunable tools for understanding how dietary carbohydrate structures are translated into microbial ecological responses and metabolic functions. Their chemical structures, particularly the anomeric configuration and linkage positions of glycosidic bonds, directly determine selective microbial utilization and downstream metabolite synthesis [2, 3]. For example, isomaltooligosaccharides linked by α‐1,6 glycosidic bonds are more effective at enriching *Bifidobacterium* and *Lactobacillus* than those with α‐1,2 or α‐1,3 configurations [4]. Oligosaccharides with α‐glycosidic bonds have been reported to increase microbial richness at fermentation endpoints under certain conditions, whereas β‐oligosaccharides specifically promote the proliferation of *Faecalibacterium prausnitzii* (*F. prausnitzii*) and *Paraprevotella clara* [3]. From a metabolic perspective, β‐1,4 oligosaccharides significantly enhance butyric acid production, whereas α‐1,4 oligosaccharides favor an increased proportion of propionic acid [3, 5]. However, most of these findings are derived from single‐time‐point observations. How defined glycosidic bonds regulate the dynamic evolution of the gut microbiota from the initial stage, drive changes in metabolite profiles, and ultimately influence host health remains insufficiently elucidated.

The structural evolution of microbial communities and the synthesis of metabolites depend on complex interaction networks involving competition and collaboration among species [6, 7]. In the nutrient‐limited gut ecosystem, competition for carbohydrate substrates is particularly important. Closely related bacteria can display strain‐specific preferences for oligosaccharides, and overlapping substrate preferences can reshape interspecies competition. For example, *Lacticaseibacillus paracasei* YT170, which harbors a higher copy number of α‐1,6‐glucosidase genes, preferentially degrades α‐1,6 glycosidic linkages in isomaltooligosaccharides and suppresses the growth of *Lactiplantibacillus plantarum* R22 [8]. In parallel, metabolic cross‐feeding represents a key cooperative mode in complex carbohydrate utilization. During the degradation of substrates such as human milk oligosaccharides, primary metabolites including acetic acid can be further converted into butyric acid by species such as *F. prausnitzii* and *Roseburia intestinalis* [9, 10]. These interactions collectively constitute a highly specialized metabolic system, within which the initial responders that recognize and utilize the primary substrate can play a pivotal role in shaping subsequent community structure and metabolic outputs. At the molecular level, the advantage of a responder depends on whether it can recognize, transport, and cleave a defined glycosidic bond. These steps are mediated by carbohydrate transporters, substrate‐binding proteins, glycoside hydrolases, and glycoside phosphorylases. Therefore, model‐based identification of microbial responders should be complemented by functional and molecular evidence to determine whether the nominated taxa possess the capacity to access the relevant glycosidic bonds in the community context.

Nevertheless, current research faces several limitations that restrict both mechanistic understanding and rational prebiotic design. First, many studies emphasize endpoint characterization and lack systematic analysis of the temporal process from structural input to microbial dynamics and functional output [11]. Second, key functional bacteria are often inferred from endpoint dominance, which can overlook early responders that initiate substrate utilization and trigger downstream metabolic shifts [12]. Third, the molecular mechanisms by which oligosaccharides with defined glycosidic bonds are recognized, transported, and cleaved remain insufficiently characterized in complex communities. Finally, analyses based only on relative abundance are susceptible to compositional bias and cannot accurately reflect numerical changes in microbial populations [13, 14]. These gaps make it difficult to convert functional oligosaccharide screening into structure‐guided rules for matching defined substrates with microbial targets and metabolic outputs.

Recent methodological advances now allow carbohydrate‐driven microbiome responses to be examined at ecological and molecular resolution. Absolute quantitative profiling improves the interpretation of community dynamics by moving beyond relative abundance. Bayesian ecological modeling can infer responder taxa with statistically supported substrate‐associated growth advantages from abundance time series while accounting for population growth and interspecies interactions [15]. Metaproteomics provides protein‐level evidence for actively expressed transporters and glycosidic bond cleavage enzymes [16], and SynComs enable inferred ecological rules to be tested in defined microbial systems [17, 18].

To apply this framework to a defined structural question, this study used five structurally defined glucose disaccharides as minimal oligosaccharide models. This design isolated glycosidic bond effects while keeping the monosaccharide composition and degree of polymerization constant. The study investigated how defined glycosidic bonds shaped gut microbial succession, which taxa displayed substrate‐associated growth advantages during this process, and whether these taxa expressed linkage‐matched molecular modules for disaccharide utilization. It further evaluated whether these ecological and molecular observations could guide the construction of SynComs with predictable substrate utilization and SCFA production patterns. Collectively, this design linked defined glycosidic bond variation to microbial responder selection, expressed substrate‐utilization modules, metabolic differentiation, and SynCom‐based functional reconstruction. These findings provide a basis for structure‐guided prebiotic design and targeted microbiome modulation.

## 2 Results and Discussion

### 2.1 Defined Glycosidic Bonds Shape Temporal Assembly of Human Gut Microbial Communities

**FIGURE 1:**
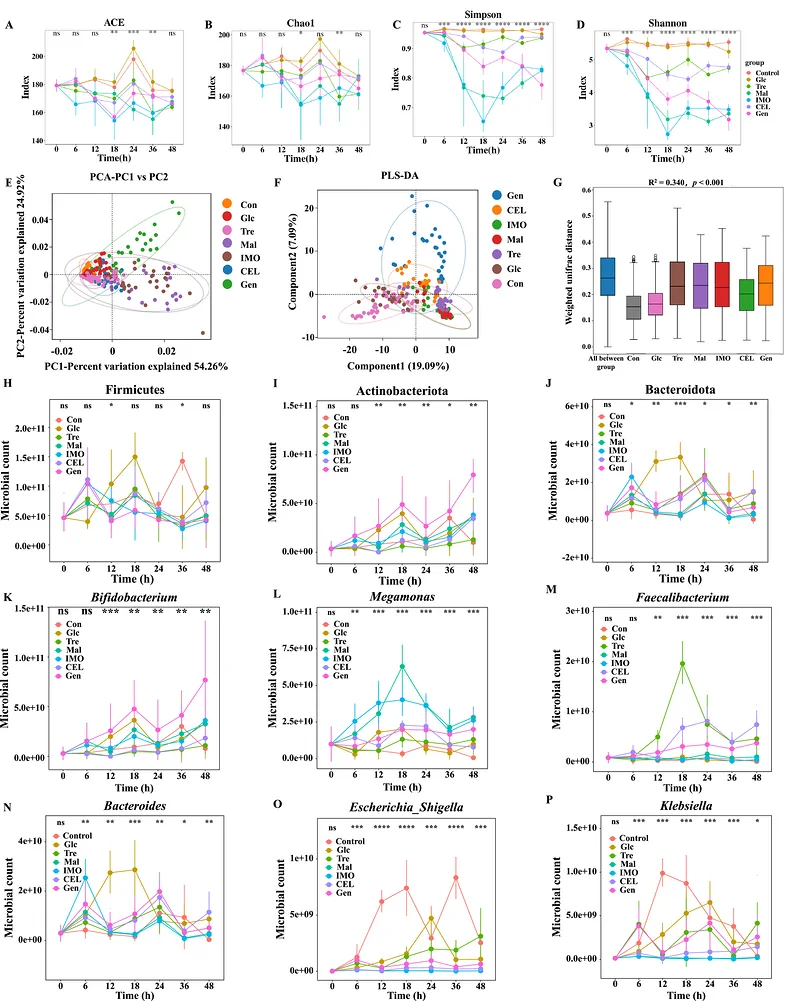
Defined glycosidic bonds shape temporal assembly and taxonomic succession of human gut microbial communities. (A–D) Time‐course changes in α‐diversity indices of human gut microbial communities during anaerobic fermentation with different carbon sources: ACE index (A), Chao1 index (B), Simpson index (C), and Shannon index (D). (E,F) β‐Diversity analysis based on PCA (E) and PLS‐DA (F). **(G)** Group‐wise distribution of weighted UniFrac distances with PERMANOVA statistics across treatment groups. (H–J) Time‐course changes in the absolute abundances of representative dominant phyla: Firmicutes (H), Actinobacteriota (I), and Bacteroidota (J). (K–P) Time‐course changes in the absolute abundances of representative genera with significant treatment‐dependent differences: *Bifidobacterium* (K), *Megamonas* (L), *Faecalibacterium* (M), *Bacteroides* (N), *Escherichia‐Shigella* (O), and *Klebsiella* (P). Con, no‐sugar control; Glc, glucose; Tre, trehalose; Mal, maltose; IMO, isomaltose; CEL, cellobiose; Gen, gentiobiose. Data are shown as mean ± SD. Statistical comparisons were performed among treatment groups at the same time point. ns, not significant; ^*^ *p* < 0.05; ^**^ *p* < 0.01; ^***^ *p* < 0.001; ^****^ *p* < 0.0001.

To investigate how defined glycosidic bonds regulate gut bacterial community succession, this study performed a 0–48 h in vitro anaerobic fermentation experiment using glucose disaccharides that differed in anomeric configuration and linkage position. α‐Diversity analysis showed that these disaccharides mainly altered community evenness, with limited effects on richness. ACE and Chao1 indices remained broadly stable across treatment groups, with only sporadic differences at individual time points, indicating that the number of detectable taxa was not substantially reshaped during the fermentation window (Figure 1A,B). In contrast, Simpson and Shannon indices decreased markedly in most disaccharide‐treated groups from 6 h onward compared with the no‐sugar and glucose controls (Figure 1C,D). The strongest reduction was observed in the gentiobiose (β‐1,6) group, which remained at the lowest evenness level at 48 h. Trehalose (α‐1,1) and cellobiose (β‐1,4) showed an early decrease followed by partial recovery, whereas maltose (α‐1,4) and isomaltose (α‐1,6) showed incomplete recovery by the end of fermentation. These results indicate that defined glycosidic bonds remodel gut bacterial communities primarily by redistributing dominance among existing community members, with gentiobiose (β‐1,6) exerting the strongest selective pressure.

β‐Diversity analysis further supported glycosidic bond structure as a major determinant of community divergence. Principal component analysis (PCA) and partial least squares discriminant analysis (PLS‐DA) showed clear separation between disaccharide‐treated communities and the two control groups, with α‐linked and β‐linked disaccharides showing distinct clustering tendencies (Figure 1E,F). The glucose group remained closer to the no‐sugar control than to most disaccharide treatments, suggesting that community divergence was not determined by monosaccharide availability alone. PERMANOVA analysis based on weighted UniFrac distance confirmed significant differences among treatment groups when all time points were considered together (R^2^ = 0.340, *p* < 0.001; Figure 1G). Within‐group PCA trajectory analysis further supported a staged succession pattern, with an early adaptation phase (0–6/12 h) in which communities remained close to the initial state, a middle enrichment phase (12–18/24 h) marked by pronounced community shifts, with gentiobiose showing a more restricted but sustained divergence pattern, and a late dynamic equilibrium phase (36–48 h) during which changes slowed but the overall structure did not return to baseline (Figure S1). The timing and magnitude of these transitions varied among glycosidic bonds.

Taxonomic analysis based on absolute abundance further resolved these bond‐dependent community shifts at the phylum and genus levels. At the phylum level, Firmicutes, initially the most abundant phylum, increased early and then showed slower temporal changes, with significant differences detected only at 12 and 36 h (*p* < 0.05; Figure 1H). Actinobacteriota progressively increased during fermentation and showed significant treatment‐dependent differences (*p* < 0.05), with the most pronounced expansion in the gentiobiose group, especially during the late phase (36–48 h) (*p* < 0.05; Figure 1I). Bacteroidota increased during the middle enrichment phase and then declined or fluctuated depending on the substrate, indicating a transient contribution to disaccharide‐driven community restructuring (Figure 1J). At the genus level, disaccharides with different glycosidic bonds enriched different carbohydrate‐responsive taxa. *Bifidobacterium* increased most strongly in the gentiobiose group and reached the highest abundance during the late phase, consistent with the marked Actinobacteriota expansion observed at the phylum level (Figure 1K). *Megamonas* diverged from 6 h onward and was most strongly promoted by maltose and isomaltose, indicating that α‐linked disaccharides preferentially supported this genus (Figure 1L). *Faecalibacterium* displayed substrate‐dependent temporal responses, with a marked transient increase in the trehalose group and a sustained late increase in the cellobiose group (Figure 1M). *Bacteroides* showed a transient enrichment pattern, particularly during early or middle fermentation depending on the substrate, suggesting participation in early substrate processing rather than stable late dominance (Figure 1N). In contrast, *Escherichia‐Shigella* and *Klebsiella* expanded more prominently in the no‐sugar and glucose controls, while remaining low in most disaccharide‐treated groups (Figure 1O,P). This pattern indicates that defined disaccharide fermentation constrains the expansion of facultative anaerobic taxa under the tested conditions.

Collectively, these results show that glycosidic bond structure programs the temporal assembly of human gut microbial communities. Despite identical glucose composition, different glycosidic bonds generated distinct patterns of community evenness, β‐diversity, phylum‐level succession, and genus‐level enrichment. This bond‐dependent assembly was characterized mainly by selective expansion of glycosidic‐bond‐responsive taxa, providing the ecological basis for subsequent modeling of responders and downstream metabolic differentiation.

### 2.2 Bayesian gLV Modeling of Absolute Time‐Series Data Identifies Glycosidic Bond‐Specific Microbial Responders

**FIGURE 2:**
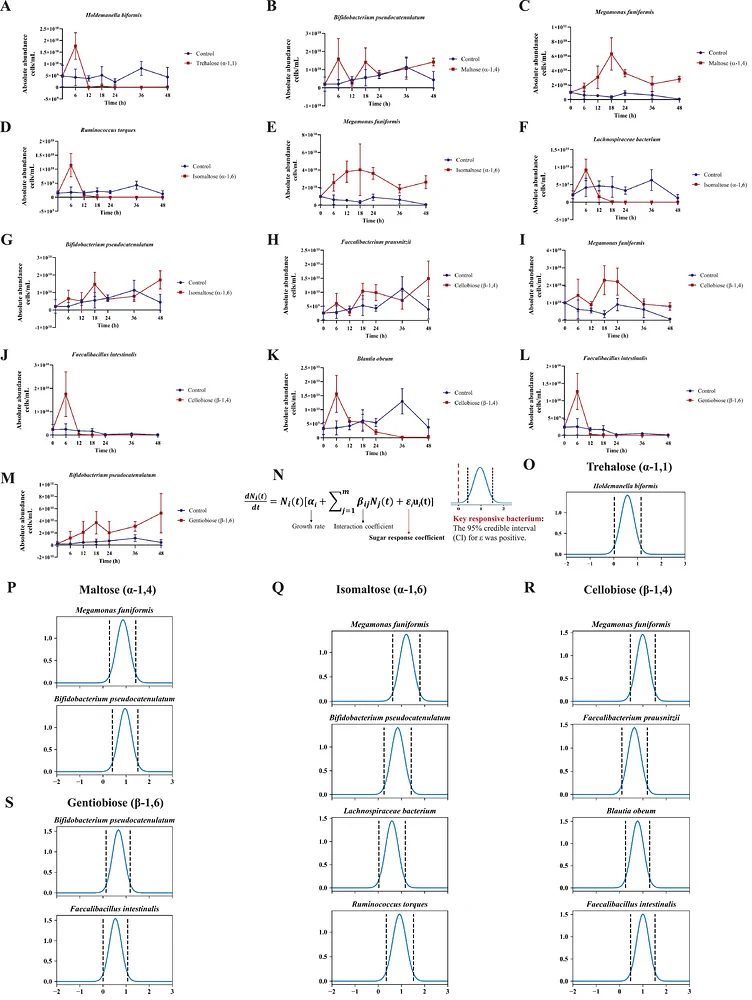
Bayesian gLV modeling identifies glycosidic bond‐specific microbial responders. (A–M) Time‐course absolute abundance dynamics of taxa nominated as model‐inferred microbial responders (red) compared with the paired no‐sugar control (blue). Panels are grouped by substrate: trehalose (α‐1,1), *H. biformis* (A); maltose (α‐1,4), *B. pseudocatenulatum* (B) and *M. funiformis* (C); isomaltose (α‐1,6), *R. torques* (D), *M. funiformis* (E), Lachnospiraceae bacterium (F), and *B. pseudocatenulatum* (G); cellobiose (β‐1,4), *F. prausnitzii* (H), *M. funiformis* (I), *F. intestinalis* (J), and *B. obeum* (K); gentiobiose (β‐1,6), *F. intestinalis* (L) and *B. pseudocatenulatum* (M). Absolute abundance is reported as spike‐in‐calibrated cell counts per mL of fermentation broth (cells/mL). (N**)** gLV model with an intervention term and the operational definition of microbial responders. (O–S) Posterior distributions of substrate response coefficients (ε) for nominated taxa under each disaccharide treatment (trehalose, O; maltose, P; isomaltose, Q; cellobiose, R; gentiobiose, S). Dashed lines indicate the posterior 95% credible interval; taxa with a posterior 95% credible interval entirely > 0 were operationally classified as model‐inferred microbial responders.

The time‐resolved community profiles suggested that defined glycosidic bonds selected distinct bacterial trajectories, but endpoint abundance alone cannot distinguish primary substrate responders from taxa that expand through downstream community interactions. To identify statistically supported responder taxa associated with bond‐dependent community assembly, a Bayesian generalized Lotka–Volterra model was applied to the absolute abundance time series of the top 20 taxa by mean absolute abundance in each disaccharide–control pair (Figure S2). The model jointly estimated intrinsic growth rates (α), interspecies interaction coefficients (β), and a substrate response coefficient (ε) for each taxon (Figure 2N). Taxa with a posterior 95% credible interval of ε entirely greater than zero were operationally classified as model‐inferred microbial responder(s), indicating a statistically supported substrate‐associated growth advantage after accounting for basal growth and interspecies interactions (Figure 2O–S, Tables S1–S5).

No model‐inferred microbial responder was identified in the glucose group, consistent with glucose serving as a broadly accessible carbon source rather than a linkage‐specific ecological filter. In contrast, each disaccharide selected a defined responder set. Under trehalose (α‐1,1) treatment, the model identified *Holdemanella biformis* as the only model‐inferred microbial responder (ε = 0.590, 95% CrI > 0; Figure 2O). Its absolute abundance increased rapidly during the first 6 h and then declined during later fermentation (Figure 2A), indicating an early responder pattern that would have been underestimated by endpoint abundance alone.

Maltose (α‐1,4) and isomaltose (α‐1,6) shared two major model‐inferred microbial responders, *Bifidobacterium pseudocatenulatum* and *Megamonas funiformis* (Figure 2P,Q). *B. pseudocatenulatum* showed positive substrate responses to both maltose (ε = 0.965) and isomaltose (ε = 0.835), while *M. funiformis* showed comparable responses to maltose (ε = 0.860) and a stronger response to isomaltose (ε = 1.210). Their time‐course profiles showed multi‐phase expansion under α‐linked disaccharides (Figure 2B,C,E,G), suggesting a partially shared responder architecture inferred by the model for α‐glycosidic bonds. Isomaltose additionally recruited *Ruminococcus torques* (ε = 0.935) and a Lachnospiraceae‐affiliated taxon (ε = 0.600), both of which showed rapid early increases followed by decline (Figure 2D,F,Q). These temporal and model‐based patterns suggest that the α‐1,6 linkage was associated with both responders shared across α‐linked disaccharides and additional early responders specific to isomaltose.

The β‐linked disaccharides produced distinct responder configurations. The model nominated *M. funiformis* (ε = 0.995), *Faecalibacterium prausnitzii* (ε = 0.645), *Blautia obeum* (ε = 0.775), and *Faecalibacillus intestinalis* (ε = 0.995) as microbial responders associated with cellobiose (β‐1,4) (Figure 2R). Their temporal dynamics suggest functional partitioning during succession. *F. intestinalis* and *B. obeum* increased rapidly at early time points and then declined, whereas *F. prausnitzii* increased progressively during the middle to late phase and became a dominant endpoint taxon (Figure 2H–K). This division between early responders and late accumulators suggests that β‐1,4 cellobiose organized a temporally ordered responder module.

For gentiobiose (β‐1,6), the model nominated a narrower responder set consisting of *B. pseudocatenulatum* (ε = 0.655) and *F. intestinalis* (ε = 0.540; Figure 2S). *B. pseudocatenulatum* showed sustained expansion and became the dominant species during late fermentation, whereas *F. intestinalis* showed an early increase followed by a decline (Figure 2L,M). This pattern is consistent with the strong reduction in community evenness observed in the gentiobiose group and supports a more restricted responder pattern centered on *B. pseudocatenulatum*.

Together, absolute‐abundance ecological modeling resolved glycosidic bond‐specific responder architectures that were not apparent from endpoint taxonomic profiles alone. α‐Linked disaccharides preferentially recruited *B. pseudocatenulatum* and *M. funiformis*, β‐1,4 cellobiose assembled a broader responder module culminating in *F. prausnitzii* expansion, and β‐1,6 gentiobiose imposed a narrower response dominated by *B. pseudocatenulatum*. The analysis therefore identified taxa with strong model‐supported substrate responses for subsequent experimental assessment of direct disaccharide utilization and expression of the corresponding substrate‐utilization machinery.

### 2.3 Microbial Responders Directly Utilize Cognate Disaccharides and Express Glycosidic‐Bond‐Compatible Transport and Cleavage Functions

The Bayesian gLV model nominated taxa with substrate‐associated growth advantages in complex communities. However, a positive response coefficient indicates a statistical association in a community context and does not by itself establish direct disaccharide utilization or explain how specific glycosidic bonds select these responders. Accordingly, the model output was used to prioritize candidate responders for subsequent functional assessment rather than as direct evidence of substrate utilization. To evaluate this ecological inference at the functional and molecular levels, monoculture validation was combined with metaproteomic profiling. Cultivable bond‐specific microbial responders were tested in monoculture using their cognate glucose disaccharides as the main carbon sources, with glucose included as a readily utilizable monosaccharide reference under otherwise identical conditions. Metaproteomic profiling was then used to determine whether linkage‐matched transport and glycosidic‐bond cleavage modules were expressed during complex‐community fermentation.

**FIGURE 3:**
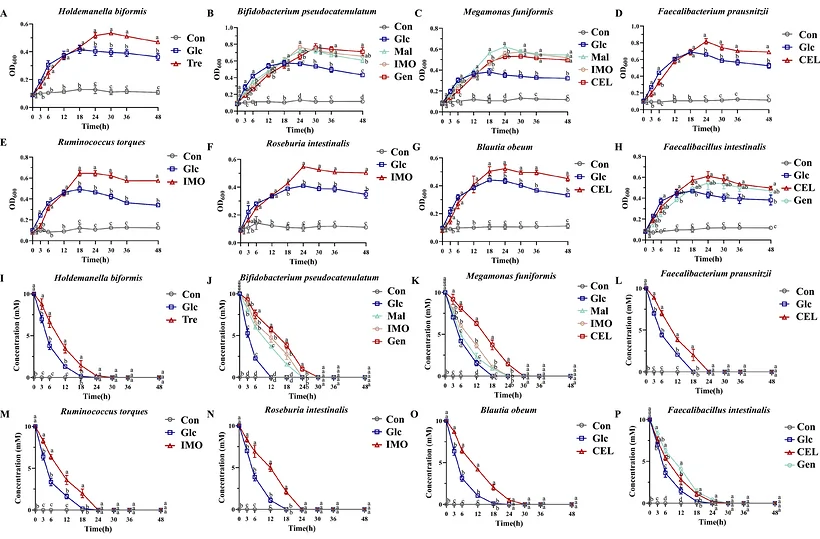
Microbial responders directly utilize cognate glucose disaccharides. (A–H) Growth curves of representative strains under the indicated substrate conditions (with no‐sugar and glucose controls as references): *Holdemanella biformis* (trehalose) (A); *Bifidobacterium pseudocatenulatum* (maltose, isomaltose, gentiobiose) (B); *Megamonas funiformis* (maltose, isomaltose, cellobiose) (C); *Faecalibacterium prausnitzii* (cellobiose) (D); *Ruminococcus torques* (isomaltose) (E); *Roseburia intestinalis* (isomaltose; used as a Lachnospiraceae representative for downstream consortium implementation) (F); *Blautia obeum* (cellobiose) (G); *Faecalibacillus intestinalis* (cellobiose, gentiobiose) (H). (I–P) Corresponding disaccharide depletion dynamics paired with strains and substrates in A–H. Statistical comparisons were performed by one‐way ANOVA with Tukey's multiple comparison test. Different letters indicate significant differences (*p* < 0.05) among groups at the same time point.

Monoculture assays confirmed that cultivable microbial responders possessed the intrinsic capacity to utilize their cognate glucose disaccharides (Figure 3). *H. biformis*, the sole α‐1,1 responder, rapidly grew on trehalose and consumed more than 99% of the substrate within 24 h, consistent with its annotated trehalose‐associated transport and intracellular cleavage components, including trehalose‐specific PTS and GH13 family proteins [19, 20]. *B. pseudocatenulatum* and *M. funiformis* efficiently utilized maltose (α‐1,4) and isomaltose (α‐1,6), with disaccharide depletion accompanying the increase in biomass, in agreement with their annotated α‐glucosidase [21, 22, 23] and oligo‐1,6‐glucosidase functions [24, 25]. Because the Lachnospiraceae‐affiliated responder could not be resolved to a cultivable species, *R. intestinalis* served as a functional proxy. The choice was based on its shared taxonomic affinity, reported α‐1,6 glycosidic‐bond utilization capacity, and butyric acid‐producing phenotype [26]. This strain also showed robust growth and near‐complete isomaltose utilization, although it may not fully reproduce the ecological behavior of the unresolved responder. In the β‐linked glucose disaccharides, *F. prausnitzii*, *B. obeum*, *M. funiformis*, and *F. intestinalis* utilized cellobiose (β‐1,4), consistent with cellobiose‐related transport and β‐glycosidic‐bond cleavage annotations [27, 28, 29, 30, 31]. *B. pseudocatenulatum* and *F. intestinalis* completely utilized gentiobiose (β‐1,6), although with slower kinetics, consistent with a more general β‐glucosidase‐associated utilization strategy [31, 32]. For comparison, glucose also supported the growth of all tested strains under identical conditions and served as a monosaccharide reference.

Across the tested strain–substrate pairs, α‐linked disaccharides were generally depleted within 18–24 h, whereas the tested β‐linked disaccharides required approximately 24–30 h. These observations suggest a linkage‐associated kinetic pattern within the tested strain panel. Gentiobiose (β‐1,6) showed the slowest utilization among the tested disaccharides. This kinetic hierarchy was consistent with the overall disaccharide utilization trends observed in complex‐community fermentation [3], suggesting that linkage‐dependent substrate preference at the single‐strain level contributes to community‐scale functional differentiation [33]. Thus, these monoculture results support the gLV inference and indicate that bond‐dependent community assembly is partly rooted in the intrinsic substrate‐use capacity of the corresponding microbial responders.

**FIGURE 4:**
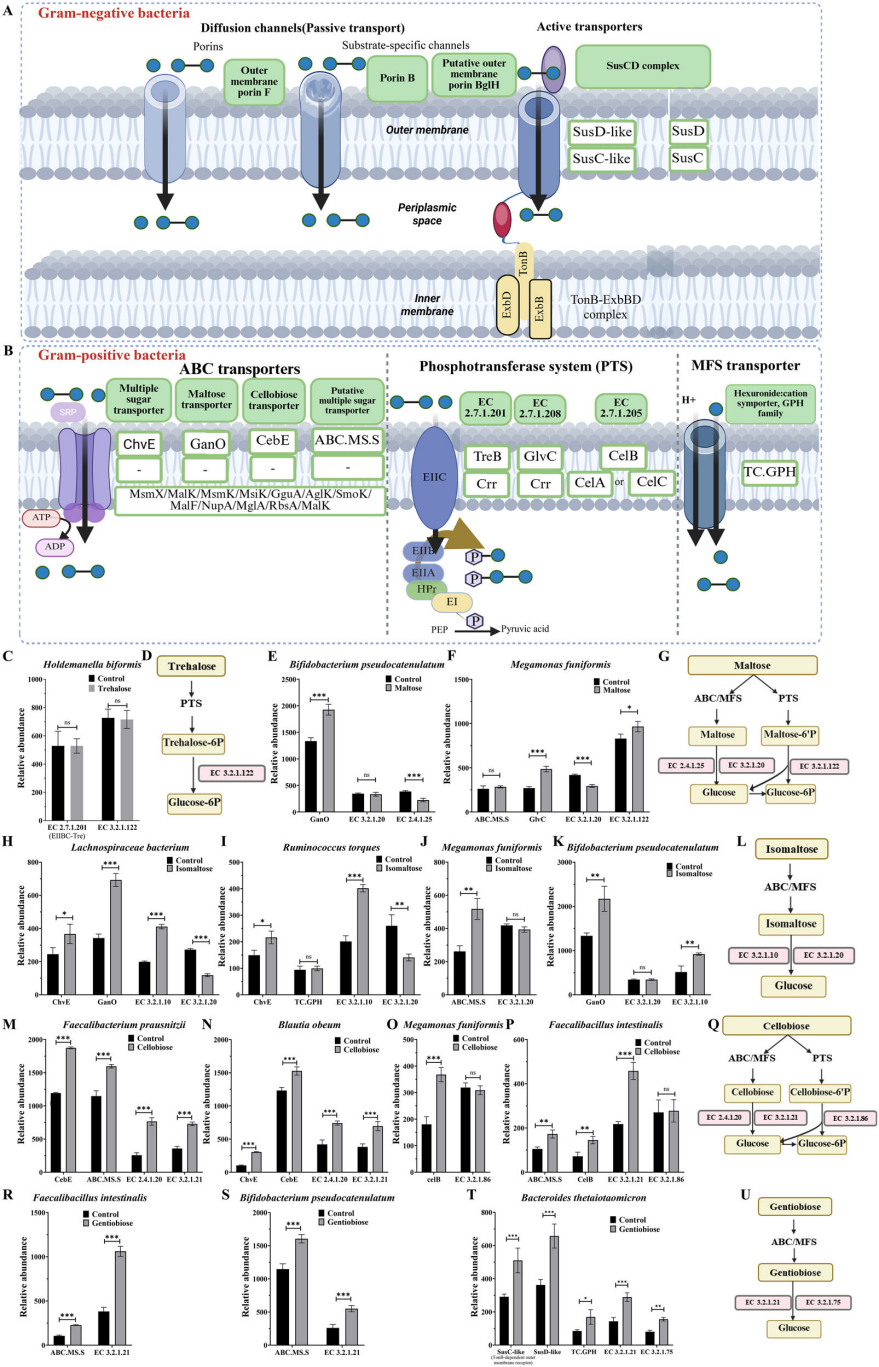
Metaproteomic analysis reveals linkage‐matched transport and glycosidic‐bond cleavage modules in microbial responders. (A) Schematic of carbohydrate uptake routes in Gram‐negative bacteria, including outer membrane channels, SusC/SusD‐like complexes, and TonB‐dependent transport. (B) Schematic of cytoplasmic membrane carbohydrate transport systems in Gram‐positive bacteria, including ABC transporters, phosphotransferase systems (PTS), and major facilitator superfamily (MFS) transporters. (C–D) Trehalose‐associated proteins in *Holdemanella biformis* and the proposed trehalose utilization route. (E–G) Maltose‐associated proteins in *Bifidobacterium pseudocatenulatum* and *Megamonas funiformis*, and the proposed maltose utilization routes. (H–L) Isomaltose‐associated proteins in Lachnospiraceae bacterium, *Ruminococcus torques*, *M. funiformis*, and *B. pseudocatenulatum*, and the proposed isomaltose utilization route. (M–Q) Cellobiose‐associated proteins in *Faecalibacterium prausnitzii*, *Blautia obeum*, *M. funiformis*, and *Faecalibacillus intestinalis*, and the proposed cellobiose utilization routes. (R–U) Gentiobiose‐associated proteins in *F. intestinalis*, *B. pseudocatenulatum*, and *Bacteroides thetaiotaomicron*, and the proposed gentiobiose utilization route. Data are shown as mean ± SD. Statistical comparisons were performed between the corresponding disaccharide treatment and control group. ns, not significant; ^*^ *p* < 0.05; ^**^ *p* < 0.01; ^***^ *p* < 0.001.

Before taxon‐resolved interpretation, global principal component analysis (PCA), partial least squares discriminant analysis (PLS‐DA), and differentially expressed protein (DEP) statistics confirmed substrate‐dependent metaproteomic variation across treatment groups (Figure S3), while overlaid data‐independent acquisition (DIA)‐based total ion chromatograms (TICs) supported chromatographic separation and reproducibility across samples (Figure S4). Metaproteomic profiling further showed that these utilization phenotypes were accompanied by expressed transport and cleavage proteins assigned to the corresponding responder taxa (Figure 4). For α‐linked glucose disaccharides, the α‐1,1, α‐1,4, and α‐1,6 modules displayed distinct responder‐specific uptake and cleavage patterns. Under trehalose (α‐1,1), proteins assigned to *H. biformis* included trehalose‐related PTS EIIABC components and phospho‐α‐glucosidase (EC 3.2.1.122), matching the predicted trehalose utilization route and the rapid early expansion of this taxon (Figure 4C,D). Although these proteins were not significantly increased relative to the paired no‐sugar control, their reproducible detection in fermentation samples supports the presence of trehalose‐related utilization machinery in *H. biformis*. Under maltose (α‐1,4), *M. funiformis* showed increased abundance of the PTS component GlvC and phospho‐α‐glucosidase (EC 3.2.1.122), whereas *B. pseudocatenulatum* showed increased abundance of the maltose‐binding protein GanO, indicating that these two α‐linked responders used different transport strategies for related substrates (Figure 4E–G). Under isomaltose (α‐1,6), *B. pseudocatenulatum*, *R. torques*, *M. funiformis*, and the Lachnospiraceae‐affiliated responder showed protein signatures related to substrate binding and α‐1,6‐directed cleavage. GanO and oligo‐1,6‐glucosidase (EC 3.2.1.10) increased in *B. pseudocatenulatum*, whereas ChvE and oligo‐1,6‐glucosidase (EC 3.2.1.10) increased in *R. torques* and the Lachnospiraceae‐affiliated responder (Figure 4H–L). These protein‐level patterns support a linkage‐matched α‐1,6 utilization module involving coordinated substrate binding and initial glycosidic‐bond cleavage.

The β‐linked glucose disaccharides displayed a different organization. Cellobiose (β‐1,4) induced a broader and more coordinated utilization module. Proteins assigned to *F. prausnitzii* and *B. obeum* included CebE/ChvE‐like substrate‐binding proteins, cellobiose phosphorylase (EC 2.4.1.20), and β‐glucosidase (EC 3.2.1.21), while *F. intestinalis* showed increased abundance of ABC.MS.S, CelB, and β‐glucosidase (EC 3.2.1.21). *M. funiformis* showed a more streamlined response dominated by increased CelB abundance (Figure 4M–Q). This pattern indicates that β‐1,4 cellobiose utilization was supported by multiple responder‐specific strategies that converged on β‐1,4 uptake and cleavage. In contrast, the gentiobiose (β‐1,6) group mainly involved ABC transport functions and general β‐glucosidase (EC 3.2.1.21) activity in *B. pseudocatenulatum* and *F. intestinalis* (Figure 4R–U). Compared with the cellobiose group, the gentiobiose group displayed fewer linkage‐directed cleavage proteins, providing a plausible molecular explanation for the slower utilization kinetics of β‐1,6 gentiobiose. Notably, although *B. thetaiotaomicron* was not identified as a model‐inferred microbial responder, gentiobiose treatment increased SusC‐like and SusD‐like outer membrane transport proteins and a β‐1,6‐specific endoglucanase (EC 3.2.1.75) assigned to this species. This result suggests a potential auxiliary role for *B. thetaiotaomicron* in β‐1,6 disaccharide processing and provides a rationale for its later inclusion as a functional complement in the gentiobiose‐targeted SynCom.

Together, monoculture validation and metaproteomics show that the nominated microbial responders possess direct disaccharide‐utilization capacity and express glycosidic‐bond‐matched substrate‐use modules. These modules connect ecological responder selection with the molecular processes of substrate recognition, disaccharide uptake, and glycosidic‐bond cleavage, supporting a molecular basis for the selective enrichment of different responder sets and their distinct utilization kinetics. These findings provide the rationale for designing responder‐centered SynComs that preserve initial disaccharide capture and enable downstream metabolic conversion.

### 2.4 Defined Glycosidic Bonds Shape Divergent Microbial SCFA and Metabolite Trajectories

**FIGURE 5:**
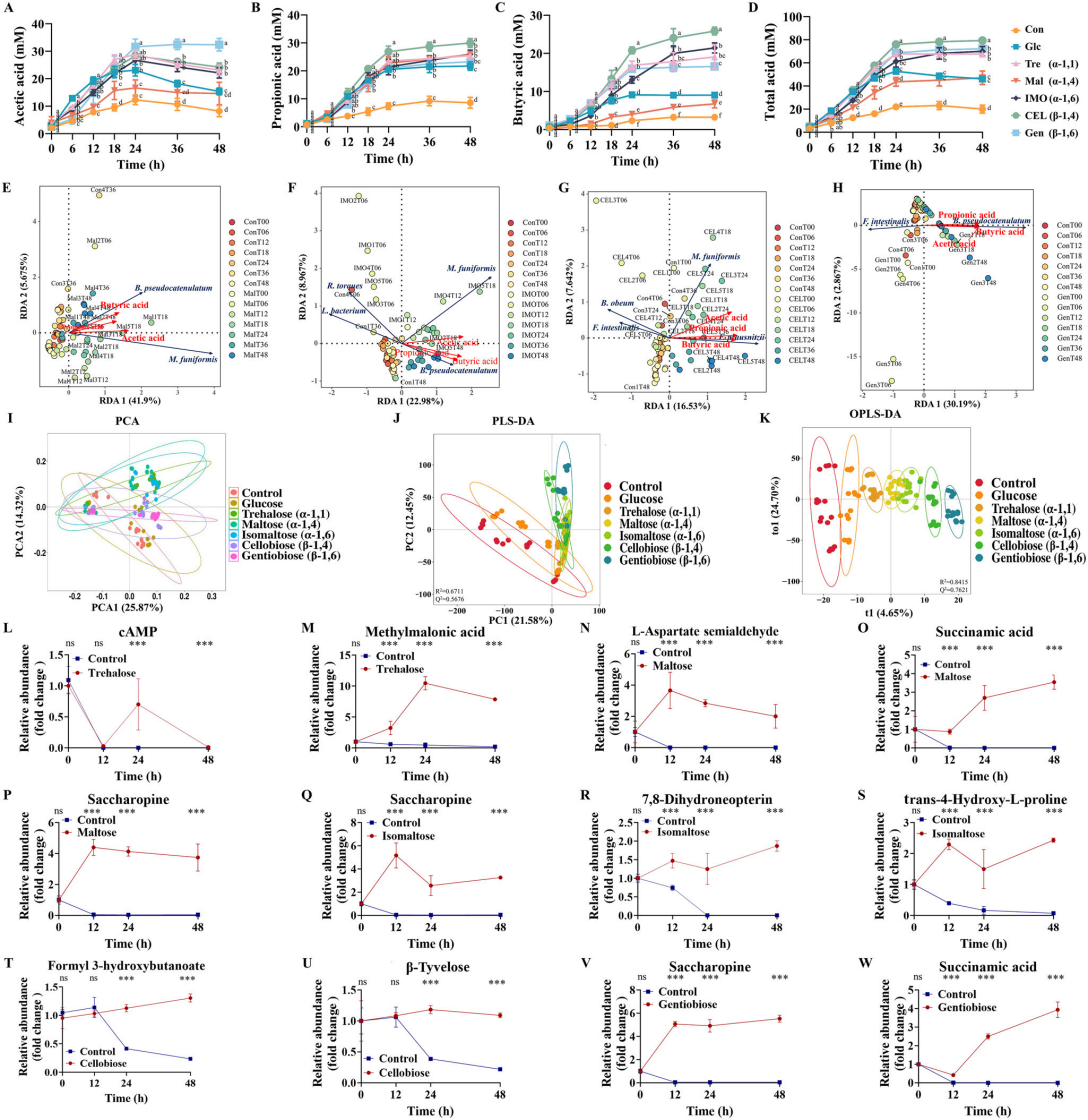
Defined glycosidic bonds shape divergent microbial SCFA and metabolite trajectories. (A–D) Time‐course concentrations of acetic acid (A), propionic acid (B), butyric acid (C), and total SCFAs (D) across treatment groups. (E–H) Redundancy analysis (RDA) of microbial‐responder absolute abundance and SCFA profiles in the maltose (E), isomaltose (F), cellobiose (G), and gentiobiose (H) groups. (I–K) Multivariate analyses of untargeted metabolomics profiles: PCA (I), PLS‐DA (J), and OPLS‐DA (K) score plots (axis labels follow the respective model outputs). (L, M) Temporal patterns of representative differential metabolites in the trehalose group. (N–P) Maltose group. (Q–S) Isomaltose group. (T–U) Cellobiose group. (V–W) Gentiobiose group. Different letters indicate significant differences among treatment groups at the same time point (one‐way ANOVA followed by Tukey's multiple comparisons; *p* < 0.05). For (L–W), values represent normalized relative abundances (peak intensities) of the indicated metabolites over time.

Consistent with the glycosidic‐bond‐dependent shifts in community structure and microbial responder selection, microbial metabolite profiles also showed linkage‐dependent divergence (Figure 5). Time‐course SCFA profiling revealed that glucose disaccharides with different glycosidic bonds generated distinct fermentation trajectories rather than converging to a uniform glucose‐derived metabolic output (Figure 5A–D). Acetic acid, a rapidly responsive SCFA [34], accumulated to the highest level in the gentiobiose (β‐1,6) group, reaching 32.50 ± 1.18 mM and remaining significantly higher than in the other groups during the late fermentation phase (*p* < 0.05; Figure 5A). In contrast, maltose (α‐1,4) showed the lowest acetic acid accumulation among the disaccharide treatments. After 24 h, acetic acid levels decreased by 5%–15% in all disaccharide groups except gentiobiose. This pattern is compatible with subsequent utilization of acetic acid and its further conversion into downstream metabolites such as butyric acid [10, 35], although the present measurements do not resolve the direction of carbon transfer.

Propionic acid showed a delayed but steady accumulation pattern (Figure 5B). At 48 h, the cellobiose (β‐1,4) group exhibited the highest propionic acid concentration, whereas the gentiobiose (β‐1,6) group remained the lowest among the disaccharide treatments (*p* < 0.05). Butyric acid displayed the strongest linkage specificity (Figure 5C). Cellobiose (β‐1,4) showed the highest butyric acid concentration, significantly exceeding all other treatments (*p* < 0.05). Isomaltose (α‐1,6) showed a delayed increase in butyric acid and ranked second to cellobiose at the late stage. Trehalose (α‐1,1) and gentiobiose (β‐1,6) showed comparable butyric acid levels, whereas maltose (α‐1,4) showed the lowest butyric acid concentration among the disaccharide groups. Total SCFAs further reflected these linkage‐dependent differences, with cellobiose showing the strongest overall acid‐producing phenotype during late fermentation (Figure 5D).

To link these SCFA trajectories to the identified responder modules, redundancy analysis (RDA) was performed using microbial‐responder absolute abundances and SCFA profiles (Figure 5E–H) [36]. The responder taxa showed clear associations with different SCFA outputs. *B. pseudocatenulatum* and *M. funiformis* were positively aligned with acetic acid, propionic acid, and butyric acid accumulation, consistent with their contribution to broad SCFA‐associated profiles. *F. prausnitzii* showed the strongest positive alignment with butyric acid in the cellobiose group, consistent with its known butyric acid‐producing capacity. By contrast, early responders such as *F. intestinalis*, *R. torques*, and *B. obeum* showed weaker or negative alignment with SCFA concentrations. Together with their rapid early expansion and subsequent decline, this pattern suggests that these taxa may be associated with early substrate utilization or intermediate production rather than sustained endpoint SCFA accumulation [37]. Thus, glycosidic bond structure influences metabolic flow by selecting responder modules with distinct temporal and functional roles [38].

Untargeted metabolomics further supported glycosidic‐bond‐specific regulation of microbial metabolic networks. Principal component analysis (PCA) and partial least squares discriminant analysis (PLS‐DA) showed that α‐linked and β‐linked disaccharide treatments formed distinct metabolic distributions, while the no‐sugar and glucose controls showed more similar profiles (Figure 5I,J). OPLS‐DA further confirmed group‐level metabolic separation (Figure 5K). Within‐group time‐course analysis showed progressive separation of metabolite profiles over 0–48 h, indicating that disaccharide‐specific metabolic states emerged dynamically during fermentation (Figure S5).

Differential metabolite analysis revealed that glycosidic bond structure affected amino acid, cofactor, and SCFA‐related metabolic pathways (Figure 5L–W, Figure S6 and Tables S7–S12). Trehalose (α‐1,1) induced sustained accumulation of methylmalonic acid, a metabolite associated with the succinate‐propionic acid pathway [39], together with a transient cAMP increase, indicating a rapid carbon‐regulatory response to α‐1,1‐linked disaccharide input (Figure 5L,M) [40]. Maltose (α‐1,4), isomaltose (α‐1,6), and gentiobiose (β‐1,6) increased saccharopine, implicating lysine‐associated metabolism potentially linked to downstream SCFA formation (Figure 5P,Q,V) [41]. Maltose (α‐1,4) and gentiobiose (β‐1,6) also increased succinamic acid, a succinate derivative, suggesting engagement of succinate‐associated metabolic routes (Figure 5O,W) [41].

Each disaccharide also showed characteristic metabolic signatures. Maltose (α‐1,4) increased L‐aspartate semialdehyde (Figure 5N). Isomaltose (α‐1,6) increased trans‐4‐hydroxy‐L‐proline and 7,8‐dihydroneopterin, a precursor for tetrahydrobiopterin biosynthesis, suggesting that the α‐1,6‐linked module may influence metabolic processes associated with redox balance and immune‐related cofactor metabolism (Figure 5R,S) [42, 43]. Cellobiose (β‐1,4) selectively increased formyl‐3‐hydroxybutanoate, a formylated derivative of 3‐hydroxybutyric acid linked to butyrate‐associated metabolism, consistent with its high butyric acid output (Figure 5T) [44]. In contrast, glucose failed to induce these linkage‐specific metabolic signatures, indicating that the structural form in which glucose units are delivered to the microbiota is critical for activating distinct community metabolic modules [45].

Collectively, these data indicate that glycosidic bond structure is associated with distinct SCFA trajectories and broader metabolite profiles. Cellobiose (β‐1,4) organized a butyric acid‐oriented module centered on *F. prausnitzii*, gentiobiose (β‐1,6) supported sustained acetic acid accumulation associated with *B. pseudocatenulatum* dominance, and isomaltose (α‐1,6) supported a broader SCFA‐associated profile.

### 2.5 Microbial Responder‐Guided SynComs Recapitulate Glycosidic Bond‐Specific Ecological Succession and Show Greater Net SCFA Accumulation

**FIGURE 6:**
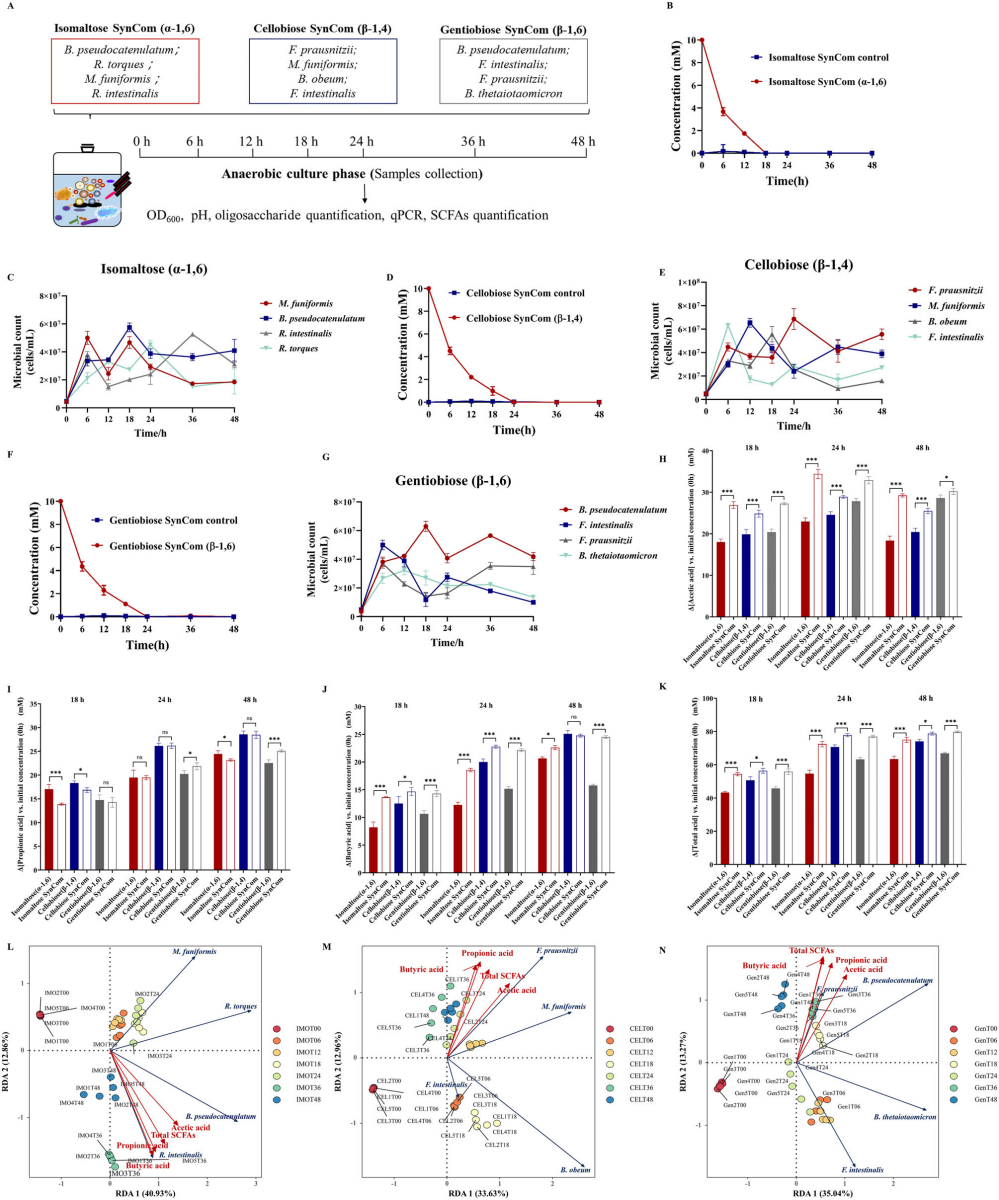
Microbial responder‐guided SynComs recapitulate glycosidic bond‐specific ecological succession and show greater net SCFA accumulation in vitro. (A) Schematic of the in vitro anaerobic fermentation process for SynComs. (B–G) Substrate concentration dynamics and strain absolute abundance succession in targeted SynComs: isomaltose degradation and strain dynamics (B, C); cellobiose degradation and strain dynamics (D, E); gentiobiose degradation and strain dynamics (F, G). (H–K) Net SCFA accumulation in SynComs versus the corresponding complex communities under the same initial substrate conditions at 18, 24, and 48 h: acetic acid (H), propionic acid (I), butyric acid (J), and total SCFAs (K). Net accumulation of each SCFA was calculated as ΔSCFA = C_t_ − C_0_ within each system. Filled bars denote SynCom fermentations and open bars denote the complex‐community fermentations under the same substrate and sampling window. Statistical significance was assessed by a two‐tailed Student's t‐test comparing SynCom versus complex communities for each substrate at each time point. (L–N) RDA of strain absolute abundance and SCFA profiles in the isomaltose‐ (L), cellobiose‐ (M), and gentiobiose‐targeted (N) SynComs. For statistical significance, ns, not significant; ^*^ *p* < 0.05; ^**^ *p* < 0.01; ^***^ *p* < 0.001.

Based on the identified microbial responders and their functional roles, three structurally streamlined SynComs were constructed for isomaltose (α‐1,6), cellobiose (β‐1,4), and gentiobiose (β‐1,6), respectively, using the core bacteria identified by the Bayesian gLV model as the design framework (Figure 6A) [18]. Strain selection followed two principles: retaining microbial responders with direct utilization capacity for the corresponding glycosidic bond, and introducing functionally complementary strains to support downstream SCFA production. The selection rationale and experimental support for each member are summarized in Table S13. In the gentiobiose SynCom, *B. thetaiotaomicron*, which is known for efficient extracellular hydrolysis of β‐1,6 glycosidic bonds [46, 47], was incorporated to facilitate β‐1,6 disaccharide processing, and *F. prausnitzii* was added to provide complementary capacity for butyric acid production [35]. In the isomaltose SynCom, *R. intestinalis* was selected as a cultivable Lachnospiraceae representative substitute for the family‐level taxon annotated as Lachnospiraceae bacterium, owing to its characterized oligo‐1,6‐glucosidase activity [26], efficient butyric acid production from carbohydrate substrates, and established relevance to host health [48]. After pre‐adaptation, all strains were mixed at equal initial OD_600_ ratios to provide comparable starting conditions for substrate‐driven succession.

In vitro fermentation confirmed that all SynComs completely and rapidly utilized their respective glucose disaccharides (Figure 6B,D,F). Isomaltose (α‐1,6) was depleted within 18 h, whereas cellobiose (β‐1,4) and gentiobiose (β‐1,6) were depleted within 24 h, consistent with the faster utilization kinetics of α‐linked disaccharides observed in monoculture assays. Disaccharide utilization was accompanied by a marked decrease in fermentation pH, and substrate‐dependent increases in community density, while no‐sugar controls showed minimal growth (Figure S7). These results indicate that microbial responder‐guided SynComs retain efficient substrate capture in a compositionally simplified system.

Time‐series analysis of absolute strain abundances revealed glycosidic bond‐specific ecological succession, suggesting a possible transition from initial resource competition to later metabolic complementarity under cognate disaccharide selection (Figure 6C,E,G) [49]. In the isomaltose SynCom (Figure 6C), the early phase was characterized by rapid proliferation of *M. funiformis*, *B. pseudocatenulatum*, and *R. torques*. During the later phase, *R. intestinalis* increased and remained abundant, consistent with a possible metabolic relay from acetic acid to butyric acid with *B. pseudocatenulatum* [10], while *M. funiformis* and *R. torques* gradually declined from their early peaks. In the cellobiose SynCom (Figure 6E), early expansion of cellobiose‐responsive strains was followed by sustained enrichment of *F. prausnitzii*, indicating a shift from early β‐1,4 disaccharide utilization to a later phase characterized by butyric acid production. In the gentiobiose SynCom (Figure 6G), *B. pseudocatenulatum* maintained a substrate‐associated growth advantage, consistent with an intracellular hydrolase‐dependent utilization strategy [50, 51]. In contrast, *B. thetaiotaomicron*, which relies on extracellular enzymes for glycan processing, gradually declined in this single‐carbon‐source environment. This decline may reflect the greater energetic demand associated with extracellular glycan‐processing systems [52]. *F. prausnitzii* increased during the later phase, consistent with the intended inclusion of complementary butyric acid‐producing capacity. The corresponding proportional strain dynamics in the in vitro SynComs are summarized in Figure S8D–F.

Substrate‐driven SynCom dynamics recapitulated the core ecological relationships observed in the complex fecal community. In the isomaltose and cellobiose modules (Figure S8A,B,D,E), early disaccharide‐responsive taxa transitioned toward late acid‐producing members, with *R. intestinalis* increasing during the later phase of the isomaltose SynCom and *F. prausnitzii* serving as the major butyric acid‐associated member in the cellobiose module. In the gentiobiose‐targeted SynCom (Figure S8C,F), the sustained enrichment of *B. pseudocatenulatum* was accompanied by an increase in *F. prausnitzii* during the later phase. These cross‐system similarities indicate that the simplified SynComs preserved a temporal sequence involving early substrate‐responsive taxa and later SCFA‐associated partners, while direct metabolite exchange among strains remains to be established.

Concomitant with efficient disaccharide utilization and structured strain succession, SCFA concentrations increased during SynCom fermentation (Figure S9). Net SCFA accumulation during the middle‐to‐late fermentation period was higher in the SynComs than in the matched complex fecal communities under the same initial disaccharide concentration and at matched sampling times, with consistent increases in acetic acid and butyric acid (Figure 6H–K). The isomaltose SynCom was characterized by rapid acetic acid and total SCFA accumulation, the cellobiose SynCom showed strong butyric acid accumulation, and the gentiobiose SynCom showed greater net accumulation of acetic acid and butyric acid than the corresponding complex community at selected matched time points.

RDA further showed that strain abundance explained a larger proportion of SCFA variation in SynComs than in the complex fecal community. The cumulative explanatory power of SynComs reached 46.59%–53.79%, exceeding that of the complex mixed community system (24.17%–33.06%) (Figure 6L–N). In the isomaltose SynCom (Figure 6L), R*. intestinalis* aligned most closely with butyric acid, while *B. pseudocatenulatum* aligned more strongly with acetic acid, consistent with a potential acetic acid‐to‐butyric acid relay but not sufficient to define direct carbon transfer. In the cellobiose SynCom (Figure 6M), F*. prausnitzii* showed strong alignment with butyric acid and total SCFAs, consistent with its known butyric acid‐producing phenotype and late‐stage enrichment. In the gentiobiose SynCom (Figure 6N), B*. pseudocatenulatum* aligned with acetic acid and total SCFAs, whereas *F. prausnitzii* aligned with butyric acid, consistent with a metabolic complementarity module between initial β‐1,6 disaccharide utilization and downstream butyric acid production. These ordination patterns describe covariation between strain abundance and SCFA output and do not demonstrate direct metabolite exchange between individual strains.

Together, these results show that microbial responder‐guided SynComs can reconstruct key features of glycosidic bond‐dependent community succession in vitro. By retaining initial disaccharide‐capturing responders and incorporating downstream conversion partners, these SynComs translate ecological inference into compositionally defined functional consortia. This approach provides a controlled platform for testing whether cognate disaccharide‐SynCom pairs retain their metabolic functions in vivo.

### 2.6 Cognate SynCom‐Disaccharide Pairs Elevate Fecal SCFAs and Alter Host‐Associated Molecular Readouts In Vivo

**FIGURE 7:**
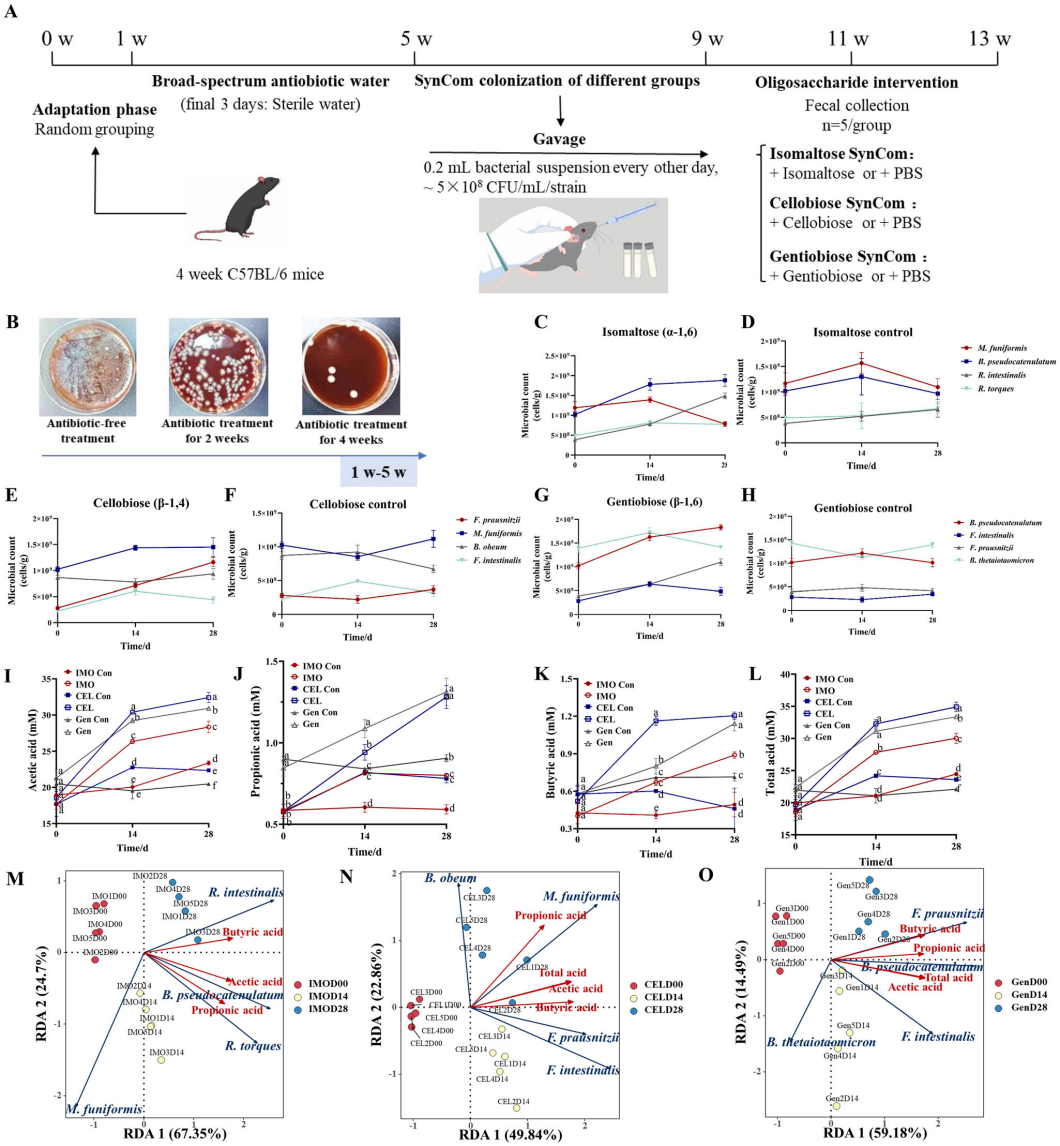
Cognate SynCom‐disaccharide pairs elevate fecal SCFAs and modulate gut barrier‐associated readouts in vivo. (A) Study design and timeline, including the adaptation phase, antibiotic treatment, SynCom colonization, and disaccharide intervention; fecal samples were collected at days 0, 14, and 28 after intervention initiation (weeks 9, 11, and 13). (B) Representative fecal culture plates before and after antibiotic treatment (weeks 1–5). (C–H) Absolute abundances (qPCR‐based) of SynCom strains in feces under disaccharide intervention versus PBS control: isomaltose group (C, D), cellobiose group (E, F), and gentiobiose group (G, H). (I–L) Fecal SCFA concentrations over time: acetic acid (I), propionic acid (J), butyric acid (K), and total SCFAs (L). (M–O) RDA linking strain abundances and SCFAs within the isomaltose (M), cellobiose (N), and gentiobiose (O) intervention groups. In I–L, statistical significance was assessed by one‐way ANOVA with Tukey's multiple comparison test; different letters indicate significant differences (*p* < 0.05) among groups at the same time point.

To assess whether the ecological and metabolic functions of microbial responder‐guided SynComs could be maintained in vivo, pseudo‐germ‐free mice were generated by broad‐spectrum antibiotic treatment and subsequently colonized with the isomaltose, cellobiose, or gentiobiose SynComs (Figure 7A,B). After SynCom colonization, mice received the cognate glucose disaccharide or PBS control for 28 days. Targeted qPCR showed that SynCom members colonized the mouse intestine and were maintained at 10^8^–10^9^ cells/g feces after the intervention period (Figure 7C–H). Strain‐specific colonization differences were observed, with *B. pseudocatenulatum*, *B. thetaiotaomicron*, *M. funiformis*, and *B. obeum* generally reaching higher fecal abundances than *R. intestinalis*, *R. torques*, *F. prausnitzii*, and *F. intestinalis*. These differences likely reflect intrinsic colonization traits, including intestinal fitness, mucus‐associated persistence, and tolerance to host‐derived ecological pressure [53].

Cognate disaccharide intervention reshaped SynCom composition in a substrate‐dependent manner. In the isomaltose SynCom, isomaltose (α‐1,6) promoted the persistence of *B. pseudocatenulatum* and increased *R. intestinalis* during the later phase of intervention, consistent with a potential downstream conversion role observed in vitro (Figure 7C). In the PBS control, *R. intestinalis* remained lower, consistent with weaker enrichment of this putative downstream converter (Figure 7D). In the cellobiose SynCom, cellobiose (β‐1,4) sustained the enrichment of *F. prausnitzii*, whereas this strain remained lower in the corresponding control group (Figure 7E,F). In the gentiobiose SynCom, gentiobiose (β‐1,6) maintained high abundance of *B. pseudocatenulatum* and promoted late enrichment of *F. prausnitzii*, while *B. thetaiotaomicron* did not become dominant at the endpoint (Figure 7G,H). These patterns indicate that cognate disaccharides were associated with substrate‐dependent changes in the composition of the introduced SynComs rather than a simple preservation of the initial inoculation ratios.

Consistent with these strain‐level shifts, cognate SynCom‐disaccharide pairs significantly elevated fecal SCFAs (Figure 7I–L). Total SCFAs accumulated during the intervention period, with the strongest increases observed in the cellobiose and gentiobiose SynCom groups (Figure 7L). SCFA composition also showed glycosidic‐bond‐associated differences. Acetic acid and propionic acid increased in all three disaccharide intervention groups, while butyric acid reached the highest level in the cellobiose SynCom group, with a 2.1‐fold higher concentration than its PBS control (Figure 7K). The gentiobiose SynCom also showed a sustained increase in butyric acid, consistent with the late enrichment and butyric acid‐associated metabolic role of *F. prausnitzii*. RDA further showed that the in vivo strain‐SCFA association patterns were broadly consistent with those observed in vitro (Figure 7M–O). In the isomaltose SynCom, *R. intestinalis* aligned with butyric acid, while *B. pseudocatenulatum* aligned more closely with acetic acid and total SCFAs. In the cellobiose SynCom, *F. prausnitzii* was associated with butyric acid and total SCFAs. In the gentiobiose SynCom, *B. pseudocatenulatum* aligned with acetic acid and total SCFAs, whereas *F. prausnitzii* aligned with butyric acid. These results indicate that the in vivo associations between strain abundance and SCFA profiles are broadly consistent with those observed in vitro.

The increase in fecal SCFAs was accompanied by changes in gut barrier‐associated and inflammatory readouts. Compared with the corresponding PBS controls, disaccharide intervention in SynCom‐colonized mice upregulated the expression of genes encoding *ZO‐1*, *Occludin*, and *Claudin‐1*, while downregulating the pore‐forming tight junction gene *Claudin‐2* (Figure S10). Serum cytokine analysis showed disaccharide‐specific reductions in selected inflammatory markers: cellobiose and gentiobiose interventions reduced TNF‐α, isomaltose and gentiobiose reduced IL‐1β, and cellobiose reduced IL‐6 (Figure S11). These changes are consistent with the immunomodulatory potential of SCFAs, particularly butyric acid [54]. Histological analysis of colon tissues showed no overt pathological damage, inflammatory cell accumulation, or epithelial disruption under the tested conditions (Figure S12). Thus, cognate SynCom‐disaccharide pairs increased fecal SCFAs and were accompanied by directional changes in barrier‐associated gene expression and selected inflammatory markers, without detectable adverse histopathology.

Together, these in vivo results show that microbial responder‐guided SynComs retain substrate‐dependent ecological and metabolic activity in the pseudo‐germ‐free mouse model. Cognate glucose disaccharides selectively shaped SynCom strain structures and elevated fecal SCFAs. These findings extend the structure‐to‐ecology‐to‐function framework from in vitro community reconstruction to a host‐associated setting, providing evidence that defined disaccharide‐SynCom pairing can be used to guide targeted gut microbiota modulation.

## 3 Conclusions

By integrating absolute time‐series ecological modeling, multiomics profiling, monoculture validation, and synthetic microbial community reconstruction, this study investigated how defined glycosidic bonds in glucose disaccharides shaped gut microbial succession, responder selection, and metabolic output. Glycosidic bond structure, including anomeric configuration and linkage position, shaped community assembly through staged succession involving adaptation, enrichment, and dynamic equilibrium. Using absolute abundance data and a Bayesian gLV model, model‐inferred microbial responders with statistically supported substrate‐associated growth advantages were identified, including taxa that were not captured by endpoint dominance alone. α‐Linked disaccharides preferentially recruited *B. pseudocatenulatum* and *M. funiformis*; β‐1,4 cellobiose supported a broader responder module that culminated in late enrichment of *F. prausnitzii*; and β‐1,6 gentiobiose produced a narrower response dominated by *B. pseudocatenulatum*. Monoculture assays and metaproteomics further provided complementary evidence that responder selection was supported by linkage‐matched molecular modules for substrate recognition, transport, and initial cleavage. These modules included PTS systems, ABC substrate‐binding proteins, oligo‐1,6‐glucosidases, β‐glucosidases, and cellobiose‐related phosphorolytic and hydrolytic enzymes.

Consistent with these ecological and molecular differences, distinct glycosidic bonds generated divergent SCFA and metabolite profiles. Gentiobiose favored sustained acetic acid accumulation; cellobiose showed the strongest butyric acid phenotype; and isomaltose rapidly promoted broad SCFA production with coordinated increases in multiple SCFA species.

Guided by these responder and metabolic patterns, we assembled model‐informed SynComs that retained direct disaccharide‐utilizing responders and incorporated complementary strains for downstream SCFA conversion. In vitro, these SynComs completely utilized their cognate disaccharides and recapitulated key temporal succession patterns progressing from early resource competition to later metabolic complementarity. Under the same initial disaccharide concentration, they also showed greater net SCFA accumulation than the matched complex communities. In pseudo‐germ‐free mice colonized with the defined SynComs, members of the cognate SynComs persisted, underwent substrate‐associated abundance shifts, and elevated fecal SCFAs (cellobiose increased butyric acid 2.1‐fold). These changes were accompanied by increased expression of selected tight junction‐related genes and reductions in selected serum cytokines, without overt histopathological damage under the tested conditions.

Several limitations should be considered. The controlled in vitro fermentation system and the mouse model established by antibiotic depletion and simplified SynCom colonization may not fully represent the complexity and interindividual variability of the human gut ecosystem. The gLV model identifies substrate‐associated population responses without resolving causal carbon flow; isotope tracing, metabolic flux analysis, and responder‐dropout experiments are therefore needed to validate cross‐feeding and responder nonredundancy. Likewise, monoculture and metaproteomic evidence do not establish gene‐level causality, which requires targeted genetic validation. Changes in tight junction‐related transcripts and serum cytokines support barrier‐associated effects but require direct permeability or epithelial repair assessments for functional confirmation. The absence of disaccharide‐only and conventional microbiota controls limits separation of SynCom‐dependent effects and extrapolation to complex gut communities. Additionally, the transferability of patterns derived from glucose disaccharides to heterooligosaccharides and structurally complex dietary fibers remains to be established.

Overall, this work supports associations among glycosidic bond structure, microbial responder dynamics, metabolic outputs, and host‐associated readouts. The combined use of ecological modeling, molecular validation, and SynCom reconstruction provides a basis for further evaluation of defined glucose disaccharide and microbial consortium pairing. Further validation across increasingly complex glycans and donor‐specific microbial communities will be required to establish the broader applicability of this strategy for structure‐guided microbiome modulation.

## 4 Experimental Section

### 4.1 Human Fecal Sample Collection

Volunteers were recruited via questionnaire distribution. From an initial screened cohort of 10 individuals, fresh fecal samples were collected from 5 healthy volunteers (18–25 years old). All participants provided written informed consent. The study protocol was approved by the Bioethics Committee of East China University of Science and Technology (Approval No. ECUST‐2023‐051) and was conducted in accordance with the principles of the Declaration of Helsinki. Inclusion criteria for volunteers were: no use of antibiotics, probiotics, or prebiotics within 3 months before sample collection; absence of gastrointestinal disorders; and consistent dietary patterns among donors as assessed by a food frequency questionnaire [3]. Fresh fecal samples were collected in sterile containers for subsequent in vitro anaerobic fermentation. Samples from the five donors were processed and fermented independently without pooling.

### 4.2 In Vitro Anaerobic Fermentation

Fecal samples from each donor were homogenized separately with sterile phosphate‐buffered saline (PBS) in an anaerobic chamber to prepare a 20% (m/v) fecal slurry. The slurry was filtered through sterile gauze to remove large particles and anaerobically activated for 2 h before use [55].

The model substrates consisted of five glucose disaccharides: trehalose (α‐1,1), maltose (α‐1,4), isomaltose (α‐1,6), cellobiose (β‐1,4), and gentiobiose (β‐1,6) (sources listed in Method S1). A glucose group and a no‐sugar control group were also included. The final concentration of each sugar was 10 mm, and the solution was sterilized by 0.22 µm filtration. The composition of the basal anaerobic medium is detailed in Method S2 [3].

Fermentation was conducted in a Bactron Anaerobic Workstation (BACTRON PLUS A35, Shel Lab, Inc., USA) under an atmosphere of 90% N_2_, 5% CO_2_, and 5% H_2_ at 37°C for 48 h. Samples were collected at 0, 6, 12, 18, 24, 36, and 48 h. Five independent biological replicates were prepared for each group at each time point.

### 4.3 Microbiome Sequencing and Analysis

Bacterial cells from fermentation broth at different time points were harvested by centrifugation. Total genomic DNA was extracted using the TGuide S96 Magnetic Soil/Stool DNA Kit (Tiangen Biotech Co., Ltd., Beijing, China). DNA concentrations were quantified and normalized before absolute abundance calibration. Seven synthetic full‐length 16S rRNA gene standards, which were distinguishable from naturally occurring microbial sequences, were mixed at a predefined copy‐number ratio of 1000:100:100:10:10:2:2. A fixed amount of the standard mixture was added to each normalized DNA sample before PCR amplification. Full‐length 16S rRNA genes were amplified by PCR using primers 27F (5'‐AGRGTTTGATYNTGGCTCAG‐3') and 1492R (5'‐TASGGHTACCTTGTTASGACTT‐3'). PCR products were confirmed by agarose gel electrophoresis, purified, quantified, normalized, and used to construct SMRTbell libraries. Qualified libraries were sequenced on the PacBio Sequel II platform (Pacific Biosciences, CA, USA) using single‐molecule real‐time (SMRT) sequencing [56].

Raw sequencing data were quality‐filtered using Trimmomatic (v0.33) and primer sequences were removed using Cutadapt (v1.9.1). Circular consensus sequences (CCS) were generated and demultiplexed using Lima (v1.7.0). Chimeric sequences were removed using UCHIME. Spike‐in reads were identified and counted using BLASTN before removal from the microbial dataset. A sample‐specific calibration curve was generated from the known copy numbers and observed reads of the seven standards. Samples were retained when spike‐in reads represented 10%–80% of total clean reads and the calibration curve had an R^2^ ≥ 0.90. Absolute taxon abundance was calculated from the corresponding calibration curve and converted to cells per milliliter after correction for sample dilution, original fermentation volume, and taxon‐specific 16S rRNA gene copy number. The same spike‐in stock, DNA input, and analytical workflow were applied across all fermentation time points. High‐quality CCS were clustered into OTUs at 97% sequence similarity using USEARCH (v10), and low‐abundance OTUs (<0.005% of total reads) were removed. Taxonomic assignment was performed using a naïve Bayes classifier against the SILVA database (v138) with a confidence threshold of 0.7. Downstream community composition and diversity analyses were performed using the BMK Cloud platform (www.biocloud.net).

### 4.4 SCFA Quantification and Untargeted Metabolomics

SCFA quantification: Gas chromatography‐flame ionization detection (GC‐FID) was used to quantify SCFA concentrations in fermentation broth and mouse feces. Sample processing followed established methods [12, 57]. Analysis was performed on an Agilent 7890 GC system (Agilent Technologies Inc., Santa Clara, CA, USA) equipped with an OV‐351 column (30 m × 0.32 mm, 1.0 µm, Dikma Technologies Inc., Beijing, China) and an FID detector, using a temperature program as described by Ji et al. [12].

Fermentation supernatants (500 µL) were extracted with methanol/acetonitrile (1:1, v/v) containing L‐2‐chlorophenylalanine as an internal standard. After protein precipitation and clarification, samples were analyzed by UPLC–MS/MS on a Q‐Exactive Plus instrument in both positive‐ and negative‐ion modes. Raw data were processed in XCalibur and metabolites were annotated by matching to HMDB and Metlin. Multivariate analyses were conducted in OmicStudio (https://www.omicstudio.cn/tool). Differential metabolites were defined using a two‐step strategy: (i) MEBA (MetaboAnalyst 6.0, https://www.metaboanalyst.ca/) to prioritize features with differential temporal patterns (top 200 by Hotelling's T^2^), and (ii) a linear model with group as the main effect and time as a covariate to identify features meeting adj. *p* < 0.05 and |log_2_FC| > 1 [58]. The final differential set was the intersection of these results.

### 4.5 Identification of Model‐Inferred Microbial Responders via Bayesian gLV Modeling

Ecological dynamics were inferred from time‐series absolute‐abundance data using a Bayesian generalized Lotka–Volterra (gLV) framework implemented following the inference strategy described by Liu et al. [15], in which an intervention term captures the net growth‐rate increment associated with a given substrate. For each disaccharide, model inference was conducted by jointly using the disaccharide‐treated time series together with its paired no‐sugar control time series. The intervention indicator u_i_(t) was defined as a binary variable (0 for control samples and 1 for disaccharide‐treated samples), enabling the substrate response coefficient ε_i_ to be interpreted as the net growth‐rate increment associated with the presence of that disaccharide, beyond basal growth α_i_ and interspecies interactions β_ij_. Posterior distributions of parameters were obtained in a Bayesian regression framework to quantify uncertainty and reduce overfitting in short time‐series settings.

For each disaccharide–control paired dataset, the top 20 species‐level taxa ranked by mean absolute abundance across all time points and biological replicates were selected for model inference. The gLV model is defined as:

(1) $$\frac{dN_{i}\left(t\right)}{dt}=N_{i}\left(t\right)\left[\alpha_{1}+\sum_{j=1}^{m}\beta_{ij}N_{j}\left(t\right)+\varepsilon_{i}u_{i}\left(t\right)\right]$$

where N_i_(t) is the absolute abundance of taxon i at time t, α_i_ is the intrinsic growth rate, β_ij_ is the interaction coefficient of taxon j on taxon i, ε_i_ is the response coefficient of taxon i to the disaccharide intervention, and u_i_(t) is the intervention indicator function.

A taxon was operationally classified as a model‐inferred microbial responder to a specific disaccharide if the posterior 95% credible interval of its estimated response coefficient ε_i_ was entirely greater than zero, indicating a statistically robust substrate‐associated growth advantage after accounting for interspecies interactions [15].

### 4.6 Bacterial Strain Cultivation and Monoculture Disaccharide Utilization Assays

Nine human‐derived gut bacterial strains used in this study—*Holdemanella biformis* (*H. biformis*, DSM 3989), *Megamonas funiformis* (*M. funiformis*, DSM 19343), *Bifidobacterium pseudocatenulatum* (*B. pseudocatenulatum*, DSM 20438), *F. prausnitzii* (DSM 107838), *Ruminococcus torques* (*R. torques*, ATCC 27756), *R. intestinalis* (DSM 14610), *Blautia obeum* (*B. obeum*, DSM 25238), *Bacteroides thetaiotaomicron* (*B. thetaiotaomicron*, VPI 5482), and *Faecalibacillus intestinalis* (*F. intestinalis*, KCTC 15631)—were purchased from Ningbo Mingzhoubio Co., Ltd. (Ningbo, China). Strain identifiers and preculture media are listed in Table S14. Strains were revived and subcultured anaerobically (37°C, 80% N_2_, 10% H_2_, 10% CO_2_) in Reinforced Clostridial Medium or Chopped Meat Carbohydrate Broth, with detailed media compositions described in Methods S3 and S4.

To shorten the lag phase, corresponding glucose disaccharides (sterile‐filtered) were added to the subculture media for pre‐adaptation before the formal experiments [59, 60]. Finally, bacterial cells at the expected exponential growth phase (approximately 12–18 h) were collected and washed three times with pre‐reduced basal anaerobic medium. The washed cells were resuspended in basal anaerobic medium and inoculated into media containing either 10 mm of the corresponding disaccharide or 10 mM glucose as a monosaccharide reference (initial OD_600_ ≈ 0.1). Parallel no‐added‐sugar controls were prepared using the same washed inocula and incubated under otherwise identical conditions. OD_600_ was monitored in the disaccharide‐containing cultures, glucose reference groups, and no‐added‐sugar controls, and residual sugar concentrations were monitored over 0–48 h. Residual sugar concentrations were detected using the 3,5‐dinitrosalicylic acid method or a trehalose‐specific assay kit (Jiangsu Aidisheng Biological Technology Co., Ltd., Yancheng, China) [3].

### 4.7 Metaproteomic Analysis of Disaccharide Transport and Cleavage Modules

Fermentation samples collected at 18 h were used for metaproteomic analysis to capture representative protein expression during active disaccharide fermentation. Samples were freeze‐dried, lysed, ground, sonicated, and centrifuged to collect protein‐containing supernatants. Protein concentrations were determined using a BCA protein assay kit. Equal amounts of protein were reduced, alkylated, digested with trypsin, desalted, and quantified before data‐independent acquisition (DIA) mass spectrometry.

LC‐MS/MS analysis was performed using a Vanquish Neo nanoUHPLC system coupled to an Orbitrap Astral mass spectrometer. Total ion chromatograms were inspected to assess chromatographic stability and technical reproducibility across biological replicates. Raw MS files were processed using Spectronaut 19. Database searching was performed against the UniProt database with trypsin specified as the digestion enzyme and up to two missed cleavages allowed. Carbamidomethylation of cysteine was set as a fixed modification, while methionine oxidation and protein N‐terminal acetylation were set as variable modifications. False discovery rates at the peptide and protein levels were controlled at ≤1%. A total of 15 696 peptides were identified and assembled into 6422 nonredundant protein groups. Protein intensities were normalized and quantified using the MaxLFQ algorithm.

Protein groups with more than 70% missing values across all samples were removed. Protein groups detected in more than 30% of samples in at least one group were retained, and remaining missing values were imputed within groups using the k‐nearest‐neighbor method. Peptide‐to‐protein inference was performed using IDPicker parsimony analysis. Taxonomic assignment was based on the source‐organism annotations of the retained protein entries, and ambiguous protein groups shared across multiple taxa were assigned to the lowest supported taxonomic level and excluded from species‐specific interpretation.

Metaproteomic interpretation focused on proteins assigned to gLV‐nominated microbial responders or functionally relevant bacteria involved in disaccharide recognition, transport, glycosidic‐bond hydrolysis, or phosphorolysis. Differentially expressed proteins were defined using FDR < 0.05 and |log_2_FC| ≥ 1.

### 4.8 Construction and In Vitro Fermentation of SynComs

Based on the gLV model inferences, three SynComs were constructed: the isomaltose SynCom (*B. pseudocatenulatum*, *R. torques*, *M. funiformis*, *R. intestinalis*), the cellobiose SynCom (*F. prausnitzii*, *M. funiformis*, *B. obeum*, *F. intestinalis*), and the gentiobiose SynCom (*B. pseudocatenulatum*, *F. intestinalis*, *F. prausnitzii*, *B. thetaiotaomicron*). As described in Section 2.5, individual strains were cultured to mid‐log phase, mixed at equal OD_600_ ratios, and inoculated into media containing the corresponding disaccharide (initial total OD_600_ ≈ 0.1) [61, 62]. The SynComs were then anaerobically cultured for 48 h, with periodic sampling for analysis of community composition and metabolic products.

### 4.9 SynCom Colonization and Cognate Disaccharide Intervention in Pseudo‐Germ‐Free Mice

Animals and ethics: All animal experimental procedures were approved by the Animal Ethics Committee of East China University of Science and Technology (Ethics No.: ECUST‐2023‐051) and conducted in strict accordance with the *Guide for the Care and Use of Laboratory Animals*. To avoid potential interference from the estrous cycle and to ensure consistent model performance, this study used 4‐week‐old SPF‐grade male C57BL/6J mice (Certificate No.: 20230004021373, License No.: SCXK 2023‐0004) purchased from Shanghai JieSiJie Laboratory Animal Co., Ltd. (Shanghai, China). Mice were housed under standard specific pathogen‐free conditions with free access to irradiated sterilized feed and sterile water, and were acclimatized for 1 week before experimentation [63]. A total of 40 mice entered the antibiotic depletion phase.

Construction of a pseudo‐germ‐free mouse model colonized with SynComs: Established methods were followed with slight modifications [64]. Briefly, mice were administered an antibiotic cocktail in drinking water for 4 weeks to deplete endogenous microbiota. The cocktail contained ampicillin (1 g/L), vancomycin hydrochloride (0.5 g/L), neomycin sulfate (1 g/L), and metronidazole (1 g/L). Antibiotic water was freshly prepared and replaced every 2–3 days. Following antibiotic treatment, surviving mice were randomly allocated to three SynCom colonization groups and gavaged with the respective SynComs described in Section 4.8. The gavage dose for each strain was approximately 5 × 10^8^ CFU/time, administered every 2 days (0.2 mL/time) for 4 weeks. Preintervention attrition occurred during antibiotic treatment and SynCom colonization.

Glucose disaccharide intervention: Following successful colonization, mice in each colonization group were further randomized into intervention and control subgroups (n = 5). The intervention group received daily gavage with the corresponding disaccharide (350 mg·kg^−^^1^·day^−^^1^), while the control group received an equal volume of PBS for 4 weeks [64]. Fecal samples were collected on days 0, 14, and 28 post‐intervention for SCFA determination and microbial community analysis. Colon tissue and blood samples were collected at the end of the experiment for further study.

### 4.10 Absolute Quantification of SynCom Strains

DNA from fermentation broth and mouse fecal samples was extracted using the TIANamp Stool DNA Kit (Tiangen Biotech Co., Ltd., Beijing, China). Absolute quantification was performed using a standard curve‐based qPCR method. Species‐specific primers were synthesized (sequences listed in Table S15B), and genomic DNA from pure strains was amplified to construct standard plasmids (detailed procedure in Method S5). A standard curve was generated using 10‐fold serial dilutions, establishing a regression equation between the Ct value and the log_10_ copy number (R^2^ ≥ 0.99). Amplification plots and standard curves for all strains are shown in Figure S13. The number of target strain cells in each sample, corrected for genome copy number, was estimated by qPCR and expressed as “cells/g feces” or “cells/mL fermentation broth”. Because conventional qPCR quantifies total target bacterial DNA, these estimated cell abundances did not distinguish viable bacterial cells from non‐viable cells or residual bacterial DNA.

### 4.11 Colon Tissue RNA Extraction and Gut Barrier Gene Expression Analysis

Total RNA was extracted from colon tissue using the TransZol Up Plus RNA Kit (Beijing TransGen Biotech Co., Ltd., Beijing, China) and reverse‐transcribed into cDNA using the Evo M‐MLV RT Mix Kit with gDNA Clean for qPCR Ver.2 (Nanjing Vazyme Biotech Co., Ltd., Nanjing, China). qPCR analysis was performed using ChamQ SYBR Color qPCR Master Mix (Nanjing Vazyme Biotech Co., Ltd., Nanjing, China). Primer sequences for the gut barrier‐related genes (*ZO‐1*, *Occludin*, *Claudin‐1*, and *Claudin‐2*) and *β‐actin* are listed in Table S15C. Relative gene expression was calculated using the 2^(‐ΔΔCt)^ method, normalized to β‐actin.

### 4.12 Mouse Gut Histological Analysis and Serum Inflammatory Cytokine Detection

Histology: Colon tissue was fixed in 4% paraformaldehyde, embedded in paraffin, sectioned, and stained with hematoxylin and eosin (H&E) for observation under a microscope [65].

Inflammatory cytokines: Serum concentrations of TNF‐α, IL‐1β, and IL‐6 were detected using commercial ELISA kits following the manufacturer's instructions (Shanghai Jiajun Biotechnology Co., Ltd., Shanghai, China).

### 4.13 Statistical Analysis

All measurement data were presented as the mean ± standard deviation. Comparisons between two groups were performed using Student's t‐test. Differences among multiple groups were analyzed by one‐way analysis of variance (ANOVA) followed by Tukey's multiple comparison test. A *p*‐value < 0.05 was considered statistically significant. For time‐course experiments, group comparisons were performed at each sampled time point unless otherwise specified.

## Author Contributions

**Xiaoxuan Lu**: investigation, methodology, validation, formal analysis, data curation, visualization, software, writing – original draft. **Jiaqi Zou, Geng Han**: investigation, validation, writing – review & editing. **Mengyao Zhao, Ting Luo**: conceptualization, writing – review & editing. **Xiangru Feng, Liangliang Zhu, Yijia Chen**: validation, writing – review & editing. **Xiaoguo Ji, Jiayang Jin**: conceptualization, methodology, funding acquisition, resources, writing – review & editing. **Liming Zhao**: conceptualization, methodology, funding acquisition, project administration, resources, writing – review & editing.

## Ethics Statement

The study involving human participants and animals was approved by the Bioethics Committee of East China University of Science and Technology (Approval No.: ECUST‐2023‐051). All human participants provided written informed consent. All procedures were conducted in accordance with the relevant guidelines.

## Conflicts of Interest

The authors declare no conflicts of interest.

## Supporting information




**Supporting File**: advs77488‐sup‐0001‐SuppMat.docx.

## Data Availability

Data are available on reasonable request to the corresponding author.
