# Supplementary material for: Oligosaccharides With Defined Glycosidic Bonds Shape Gut Microbial Succession and Metabolism Via Bond‐Specific Microbial Responders
Source: Adv Sci (Weinh). 2026 Sep 1:e77488. Online ahead of print. doi: 10.1002/advs.77488 (PMC13534784; doi:10.1002/advs.77488)
Supplement: Supplementary file 1 [file ADVS-9999-e77488-s001.docx]

Supporting Information

**Oligosaccharides with defined glycosidic bonds shape gut microbial succession and metabolism via bond-specific keystone responders**

*Xiaoxuan Lu, Jiaqi Zou, Geng Han, Mengyao Zhao, Ting Luo, Xiangru Feng, Liangliang Zhu, Yijia Chen, Xiaoguo Ji*, Jiayang Jin*, Liming Zhao**

A. B. C. D. Xiaoxuan Lu, Jiaqi Zou, Geng Han, Mengyao Zhao, Ting Luo, Xiangru Feng, Liangliang Zhu, Yijia Chen

A. B. C. D. Xiaoguo Ji

E-mail: [jixiaoguo@ecust.edu.cn](mailto:jixiaoguo@ecust.edu.cn)

A. B. C. D. Jiayang Jin

E-mail: jinjiayang@ecust.edu.cn

A. B. C. D. Liming Zhao

E-mail: [zhaoliming@ecust.edu.cn](mailto:zhaoliming@ecust.edu.cn)

**Affiliations:**

A State Key Laboratory of Bioreactor Engineering, East China University of Science and Technology, Shanghai, China

B Shanghai Frontiers Science Center of Optogenetic Techniques for Cell Metabolism, School of Pharmacy, East China University of Science and Technology, Shanghai, China

C Shanghai Collaborative Innovation Center for Biomanufacturing Technology (SCICBT), Shanghai, China

D ECUST Medical-Engineering Integration Innovation Center, Shanghai, China

# **Supplementary Methods**

## **S1.1 Reagents and materials**

All chemical reagents, including L-cysteine, hemin, and bile salts, were purchased from Thermo Fisher Scientific (MA, USA). Methanol, acetonitrile, and ethyl acetate were obtained from Adamas (Switzerland). Short-chain fatty acid (SCFA) standards (acetic acid, propionic acid, and butyric acid) were purchased from Aladdin Biochemical Technology Co., Ltd. (Shanghai, China). Reinforced Clostridial Medium (RCM) was purchased from Haibo Biotechnology (HB0136-3, China). Disaccharide substrates—trehalose (α-1,1) and β-lactose (β-1,4) from Aladdin; maltose (α-1,4) from Sinopharm Chemical Reagent Co., Ltd. (Shanghai, China); isomaltose (α-1,6) from Picasso (Shanghai, China); cellobiose (β-1,4) from Macklin (Shanghai, China); and gentiobiose (β-1,6) from Yuanye (Shanghai, China)—were all of high purity (95%–99%). All other chemicals and solvents were of analytical grade or met standard experimental requirements.

## **S1.2 Basal anaerobic medium formulation**

The medium contained (per liter): 2.0000 g yeast extract, 2.0000 g peptone, 2.0000 g NaHCO₃, 0.5000 g L-cysteine hydrochloride, 0.5000 g bile salts, 2.0000 g Tween 80, 0.1000 g NaCl, 0.0400 g K₂HPO₄, 0.0400 g KH₂PO₄, 0.0100 g MgSO₄·7H₂O, 0.0100 g CaCl₂·6H₂O, 0.0050 g hemin, 0.0010 g vitamin K₁, pH 7.0.

## **S1.3 Chopped Meat Carbohydrate Broth (CMC) formulation**

The medium contained (per liter): 30.0000 g peptone, 10.0000 g beef extract, 5.0000 g yeast extract, 5.0000 g dipotassium phosphate, 0.5000 g L-cysteine·HCl, 0.0005 g resazurin, 1.0000 g starch, 4.000 g glucose, 1.0000 g cellobiose, 1.000 g maltose, 0.0050 g hemin, 0.0500 g vitamin K₁, pH 7.0.

## **S1.4 Reinforced Clostridial Medium (RCM) formulation**

The medium contained (per liter): 10.0000 g peptone, 10.0000 g beef extract powder, 3.0000 g yeast extract powder, 5.0000 g glucose, 1.0000 g soluble starch, 5.0000 g sodium chloride, 3.0000 g sodium acetate, 0.5000 g L-cysteine hydrochloride, 0.5000 g agar, pH 6.8.

## **S1.5 Standard plasmid preparation workflow**

For each target strain, specific primers (sequences listed in **Table S14B**) were used to amplify a genomic fragment from purified bacterial genomic DNA via PCR. The PCR product was gel-purified and ligated into the pMD19-T Vector (TaKaRa, Japan). The ligation product was transformed into *E. coli* competent cells. Putative positive clones were initially selected via blue-white screening on LB plates containing X-gal and IPTG. White single colonies were picked for culture expansion, and plasmids were extracted and verified by sequencing for correct insert sequence. Plasmid concentration was measured using a NanoDrop 2000, and the copy number concentration was calculated using the formula: Copies/μL = [Plasmid concentration (ng/μL) × 10⁻⁹ × 6.02 × 10²³] / [Total plasmid molecular weight (bp) × 660].

The standard plasmid was subjected to 10-fold serial dilutions. A standard curve relating the C_t_ value to the logarithm of the copy number (log₁₀ copies) was constructed via qPCR amplification, fitting a regression equation (y = ax + b, where y is the C_t_ value and x is the log₁₀ copy number) with a verified correlation coefficient (R² ≥ 0.99). The absolute copy number of the target strain in a sample was derived by substituting its C_t_ value into the standard curve regression equation. Each sample was analyzed with three technical replicates, and results were initially expressed as "copies/g feces" or "copies/mL fermentation broth." This value was subsequently corrected based on the target gene copy number per genome for that strain (see **Table S14**) to yield the final result expressed as "cells/g feces" or "cells/mL fermentation broth," representing the number of target bacterial cells per gram of feces or per milliliter of fermentation broth.

# **Supplementary Figures (Figure S1-S10)**

**Figure S1. Temporal PCA trajectories of gut microbial communities under each carbon source treatment.**

**A–F** Within group PCA trajectory analysis of gut microbial community structure during 0 to 48 h anaerobic fermentation in the glucose (A), trehalose (B), maltose (C), isomaltose (D), cellobiose (E), and gentiobiose (F) groups. Green dashed boxes indicate the early adaptation phase, and red dashed boxes indicate the late dynamic equilibrium phase. Intermediate time points represent the enrichment phase. Ellipses indicate 95% confidence intervals.

**Figure S2. Absolute abundance dynamics of the top 20 species level taxa across carbon source treatments.**

**A–G** Absolute abundance dynamics of the top 20 species: Blank control (A); Glucose (B); Trehalose (C); Maltose (D); Isomaltose (E); Cellobiose (F); Gentiobiose (G).

**Figure S3. Global metaproteomic variation and differentially expressed proteins across disaccharide treatments.**

**A.** Principal component analysis (PCA) of metaproteomic profiles across different substrate treatments. **B.** Partial least squares discriminant analysis (PLS-DA) of metaproteomic profiles. **C.** Volcano plots showing differentially expressed proteins (DEPs) in each treatment compared with the control group. Red, blue, and gray dots indicate upregulated, downregulated, and non-significant proteins, respectively. **D.** Numbers of upregulated and downregulated DEPs in each treatment compared with the control group.

**Figure S4. DIA-based metaproteomic total ion chromatograms showing chromatographic separation and reproducibility across substrate treatments.**

Overlaid total ion chromatograms (TICs) from data-independent acquisition (DIA)-based metaproteomic analysis are shown for each substrate group. **A**. Control group. **B**. Glucose group. **C**. Trehalose group. **D**. Maltose group. **E**. Isomaltose group. **F**. Cellobiose group. **G**. Gentiobiose group.

**Figure S5. Temporal metabolomic trajectories within each carbon source treatment.**

**A**. Glucose group; **B**. Trehalose group; **C**. Maltose group; **D**. Isomaltose group; **E**. Cellobiose group; **F**. Gentiobiose group.

**Figure S6. Classification of differential metabolites induced by different carbon source treatments.**

**A-F** Class level: Glucose (A); Trehalose (B); Maltose (C); Isomaltose (D); Cellobiose (E); Gentiobiose (F). **G-L** Subclass level: Glucose (G); Trehalose (H); Maltose (I); Isomaltose (J); Cellobiose (K); Gentiobiose (L).

**Figure S7. pH and growth dynamics of targeted SynComs during *in vitro* fermentation.**

**A, B** pH profile (A) and OD₆₀₀ growth curve (B) of the isomaltose-targeted SynCom. **C, D** pH profile (C) and OD₆₀₀ growth curve (D) of the cellobiose-targeted SynCom.**E, F** pH profile (E) and OD₆₀₀ growth curve (F) of the gentiobiose-targeted SynCom.

**Figure S8. Strain proportion dynamics in complex fecal communities, *in vitro* SynComs, and SynCom colonized mice.**

**A–C** Proportion changes of keystone responders during *in vitro* fermentation of the complex native community driven by isomaltose (A), cellobiose (B), and gentiobiose (C). **D–F** Strain proportion dynamics in *in vitro* fermented SynComs targeted for isomaltose (D), cellobiose (E), and gentiobiose (F). **G–I** Strain proportion dynamics in SynCom-colonized mice under *in vivo* intervention with isomaltose (G), cellobiose (H), and gentiobiose (I).

**Figure S9. SCFA production by targeted SynComs during *in vitro* fermentation.**

**A–D** Time-course concentrations of acetic acid (A), propionic acid (B), butyric acid (C), and total SCFAs (D) in fermentation broths of the isomaltose-, cellobiose-, and gentiobiose-targeted SynComs. *p*-values were determined by one-way analysis of variance (ANOVA) with Tukey's multiple comparison test. Different letters represented a significant difference of *p* < 0.05 between the groups with multiple comparisons.

**Figure S10. Colonic barrier associated gene expression in SynCom colonized pseudo-germ-free mice after 28 days of disaccharide intervention.**

Expression levels (fold change normalized to PBS control) of: *ZO-1* (A), *Occludin* (B), *Claudin-1* (C), *Claudin-2* (D).

**Figure S11. Serum inflammatory cytokine levels in SynCom colonized pseudo-germ-free mice after 28 days of disaccharide intervention.**

**A–C** Serum concentrations of TNF-α (A), IL-6 (B), and IL-1β (C) after cognate disaccharide or PBS intervention in SynCom colonized pseudo-germ-free mice.

**Figure S12. Histological assessment of colon tissues after SynCom colonization and disaccharide intervention.**

**A–B** H&E staining of colon tissues from mice colonized with the isomaltose SynCom and treated with PBS control (A) or isomaltose (B). **C–D**, H&E staining of colon tissues from mice colonized with the cellobiose SynCom and treated with PBS control (C) or cellobiose (D). **E–F**, H&E staining of colon tissues from mice colonized with the gentiobiose SynCom and treated with PBS control (E) or gentiobiose (F). Each panel includes representative images at 40× and 200× magnification.

**Figure S13. Amplification plots and standard curves for qPCR based absolute quantification of target bacterial strains.**

**A–H,** Amplification plots and standard curves for *Bifidobacterium pseudocatenulatum* (A), *Faecalibacterium prausnitzii* (B), *Roseburia intestinalis* (C), *Ruminococcus torques* (D), *Bacteroides thetaiotaomicron* (E), *Faecalibacillus intestinalis* (F), *Megamonas funiformis* (G), and *Blautia obeum* (H).

# **Supplementary Tables (Table S1-S14)**

**Table S1. Significant parameters (Significant=TRUE, 95% CI not crossing zero) from the gLV model for gut microbial growth response and interspecies/intraspecies interactions under Trehalose treatment.**

| **Type** | **Taxa** | **Variable** | **Left** | **Right** | **Middle** | **Median** | **SNR** | **Significant** |
| --- | --- | --- | --- | --- | --- | --- | --- | --- |
| Trehalose_response | Holdemanella_biformis | epsilon__Holdemanella_biformis | 0.03 | 1.15 | 0.59 | 0.576268 | 2.075604038 | TRUE |
| pairwise_interaction | ('Megamonas_funiformis', 'Megamonas_funiformis') | beta__Megamonas_funiformis_Megamonas_funiformis | -3.38 | -0.63 | -2.005 | -1.983565 | -2.887605603 | TRUE |
| pairwise_interaction | ('Megamonas_funiformis', 'Holdemanella_biformis') | beta__Megamonas_funiformis_Holdemanella_biformis | -4.14 | -0.84 | -2.49 | -2.523915 | -3.00442277 | TRUE |
| pairwise_interaction | ('Blautia_obeum', 'Faecalibacterium_prausnitzii') | beta__Blautia_obeum_Faecalibacterium_prausnitzii | -3.18 | -0.24 | -1.71 | -1.724715 | -2.315093294 | TRUE |

**Table S2. Significant gLV model parameters for the Maltose treatment group.**

| **Type** | **Taxa** | **Variable** | **Left** | **Right** | **Middle** | **Median** | **SNR** | **Significant** |
| --- | --- | --- | --- | --- | --- | --- | --- | --- |
| Maltose_response | Megamonas_funiformis | epsilon__Megamonas_funiformis | 0.3 | 1.42 | 0.86 | 0.8621165 | 3.09432952 | TRUE |
| Maltose_response | Bifidobacterium_pseudocatenulatum | epsilon__Bifidobacterium_pseudocatenulatum | 0.41 | 1.52 | 0.965 | 0.964695 | 3.452633725 | TRUE |
| pairwise_interaction | ('Megamonas_funiformis', 'Megamonas_funiformis') | beta__Megamonas_funiformis_  Megamonas_funiformis | -2.77 | -0.25 | -1.51 | -1.50188 | -2.344347395 | TRUE |
| pairwise_interaction | ('Bifidobacterium_pseudocatenulatum', 'Megamonas_funiformis') | beta__Bifidobacterium_pseudocatenulatum_Megamonas_funiformis | -2.54 | -0.03 | -1.285 | -1.33509 | -2.040998442 | TRUE |

**Table S3. Significant gLV model parameters for the Isomaltose treatment group.**

| **Type** | **Taxa** | **Variable** | **Left** | **Right** | **Middle** | **Median** | **SNR** | **Significant** |
| --- | --- | --- | --- | --- | --- | --- | --- | --- |
| Isomaltose_response | Megamonas_funiformis | epsilon__Megamonas_funiformis | 0.62 | 1.8 | 1.21 | 1.217405 | 4.13968174 | TRUE |
| Isomaltose_response | Bifidobacterium_pseudocatenulatum | epsilon__Bifidobacterium_pseudocatenulatum | 0.25 | 1.42 | 0.835 | 0.8407105 | 2.89878163 | TRUE |
| Isomaltose_response | Lachnospiraceae_bacterium | epsilon__Lachnospiraceae_bacterium | 0.03 | 1.17 | 0.6 | 0.5922605 | 2.084470467 | TRUE |
| Isomaltose_response | _leftsquarebracket_Ruminococcus_rightsquarebracket__torques | epsilon___leftsquarebracket_Ruminococcus_rightsquarebracket__torques | 0.35 | 1.52 | 0.935 | 0.932808 | 3.206060042 | TRUE |
| pairwise_interaction | ('Megamonas_funiformis', 'Megamonas_funiformis') | beta__Megamonas_funiformis_Megamonas_funiformis | -3 | -0.43 | -1.715 | -1.74105 | -2.697231438 | TRUE |
| pairwise_interaction | ('Bifidobacterium_pseudocatenulatum', 'Megamonas_funiformis') | beta__Bifidobacterium_pseudocatenulatum_Megamonas_funiformis | -2.56 | -0.06 | -1.31 | -1.308725 | -2.10689351 | TRUE |
| pairwise_interaction | ('Lachnospiraceae_bacterium', 'Megamonas_funiformis') | beta__Lachnospiraceae_bacterium_Megamonas_funiformis | -2.79 | -0.17 | -1.48 | -1.514115 | -2.33445098 | TRUE |

**Table S4. Significant gLV model parameters for the Cellobiose treatment group.**

| **Type** | **Taxa** | **Variable** | **Left** | **Right** | **Middle** | **Median** | **SNR** | **Significant** |
| --- | --- | --- | --- | --- | --- | --- | --- | --- |
| basal_growth_rate | Megamonas_funiformis | alpha__Megamonas_funiformis | 0.13 | 1.04 | 0.585 | 0.596898 | 2.58585712 | TRUE |
| Cellobiose_response | Megamonas_funiformis | epsilon__Megamonas_funiformis | 0.46 | 1.53 | 0.995 | 0.9854795 | 3.643479069 | TRUE |
| Cellobiose_response | Faecalibacterium_prausnitzii | epsilon__Faecalibacterium_prausnitzii | 0.1 | 1.19 | 0.645 | 0.634369 | 2.298114533 | TRUE |
| Cellobiose_response | Blautia_obeum | epsilon__Blautia_obeum | 0.26 | 1.29 | 0.775 | 0.774496 | 2.930434051 | TRUE |
| Cellobiose_response | Faecalibacillus_intestinalis | epsilon__Faecalibacillus_intestinalis | 0.47 | 1.52 | 0.995 | 1.001105 | 3.793533514 | TRUE |
| pairwise_interaction | ('Megamonas_funiformis', 'Megamonas_funiformis') | beta__Megamonas_funiformis_Megamonas_funiformis | -4.15 | -1.68 | -2.915 | -2.891345 | -4.724243933 | TRUE |
| pairwise_interaction | ('Bifidobacterium_pseudocatenulatum', 'Holdemanella_biformis') | beta__Bifidobacterium_pseudocatenulatum_Holdemanella_biformis | -3.36 | -0.01 | -1.685 | -1.60705 | -1.934566851 | TRUE |
| pairwise_interaction | ('Blautia_obeum', 'Megamonas_funiformis') | beta__Blautia_obeum_Megamonas_funiformis | -2.37 | -0.18 | -1.275 | -1.289285 | -2.293809868 | TRUE |
| pairwise_interaction | ('Faecalibacillus_intestinalis', 'Megamonas_funiformis') | beta__Faecalibacillus_intestinalis_Megamonas_funiformis | -2.76 | -0.23 | -1.495 | -1.52092 | -2.431032582 | TRUE |

**Table S5. Significant gLV model parameters for the Gentiobiose treatment group.**

| **Type** | **Taxa** | **Variable** | **Left** | **Right** | **Middle** | **Median** | **SNR** | **Significant** |
| --- | --- | --- | --- | --- | --- | --- | --- | --- |
| Gentiobiose_response | Bifidobacterium_pseudocatenulatum | epsilon__Bifidobacterium_pseudocatenulatum | 0.13 | 1.18 | 0.655 | 0.669221 | 2.551323235 | TRUE |
| Gentiobiose_response | Faecalibacillus_intestinalis | epsilon__Faecalibacillus_intestinalis | 0.01 | 1.07 | 0.54 | 0.531618 | 1.996731038 | TRUE |
| pairwise_interaction | ('Megamonas_funiformis', 'Megamonas_funiformis') | beta__Megamonas_funiformis_Megamonas_funiformis | -3.92 | -0.25 | -2.085 | -2.072825 | -2.278365923 | TRUE |

**Table S6. Keystone responders identified by Bayesian gLV modeling and their putative carbohydrate transport and glycosidic bond cleavage functions.**

| **Group (Glycosidic bond)** | **Key bacterial responders** | **ε value ( 95% CI)** | **Predicted associated functional protein / enzyme** | **Reference / Accession number** |
| --- | --- | --- | --- | --- |
| **Glucose** | No |  |  |  |
|  |  |  |  |  |
| **Trehalose (α-1,1)** | *Holdemanella biformis* | 0.590 (0.030, 1.150) | PTS system trehalose-specific EIIBC component | WP_337741526.1  (National Center for Biotechnology Information. PTS system trehalose-specific EIIBC component [*Holdemanella biformis*] (NCBI Reference Sequence: WP_337741526.1). Bethesda (MD): National Library of Medicine (US); 2024 [cited 2025 Dec 20]. Available from: <https://www.ncbi.nlm.nih.gov/protein/WP_337741526.1>) |
|  |  |  | Glycoside hydrolase family 13 protein | WP_302129760.1  (National Center for Biotechnology Information. glycoside hydrolase family 13 protein [*Holdemanella biformis*] (NCBI Reference Sequence: WP_302129760.1). Bethesda (MD): National Library of Medicine (US); 2025 [cited 2025 Dec 20]. Available from: <https://www.ncbi.nlm.nih.gov/protein/WP_302129760.1>) |
|  |  |  |  |  |
| **Maltose (α-1,4)** |  |  |  |  |
|  | *Bifidobacterium pseudocatenulatum* | 0.965 (0.410,1.520) | Putative alpha-1,4-glucosidase | Li W, Liang H, He W, Gao X, Wu Z, Hu T, et al. Genomic and functional diversity of cultivated *Bifidobacterium* from human gut microbiota. Heliyon. 2024;10(5):e27270；  BAR04414.1  (National Center for Biotechnology Information. Putative alpha-1,4-glucosidase [*Bifidobacterium pseudocatenulatum* DSM 20438 = JCM 1200 = LMG 10505] (GenBank: BAR04414.1). Bethesda (MD): National Library of Medicine (US); 2016 [cited 2025 Dec 20]. Available from: <https://www.ncbi.nlm.nih.gov/protein/BAR04414.1>) |
|  | *Megamonas funiformis* | 0.860 (0.300,1.400) | Alpha-glucosidase | QIB61021.1  (National Center for Biotechnology Information. alpha-glucosidase [*Megamonas funiformis*] (GenBank: QIB61021.1). Bethesda (MD): National Library of Medicine (US); 2020 [cited 2025 Dec 20]. Available from: <https://www.ncbi.nlm.nih.gov/protein/QIB61021.1>) |
|  |  |  |  |  |
| **Isomaltose (α-1,6)** |  |  |  |  |
|  | *Bifidobacterium pseudocatenulatum* | 0.835 (0.250,1.420) | Oligo-1,6-glucosidase | BAR04460.1  (National Center for Biotechnology Information. oligo-1,6-glucosidase [*Bifidobacterium pseudocatenulatum* DSM 20438 = JCM 1200 = LMG 10505] (GenBank: BAR04460.1). Bethesda (MD): National Library of Medicine (US); 2016 [cited 2025 Dec 20]. Available from: <https://www.ncbi.nlm.nih.gov/protein/BAR04460.1>) |
|  | *Ruminococcus torques* | 0.935 (0.350,1.520) | Glycosidases | CBL25404.1  (National Center for Biotechnology Information. Glycosidases [*[Ruminococcus] torques* L2-14] (GenBank: CBL25404.1). Bethesda (MD): National Library of Medicine (US); 2015 [cited 2025 Dec 20]. Available from: <https://www.ncbi.nlm.nih.gov/protein/CBL25404.1>) |
|  | *Megamonas funiformis* | 1.210 (0.620,1.800) | Oligo-1,6-glucosidase | CDB93667.1  (National Center for Biotechnology Information. Glycosidases [*Megamonas funiformis* CAG:377] (GenBank: CDB93667.1). Bethesda (MD): National Library of Medicine (US); 2013 [cited 2025 Dec 20]. Available from: <https://www.ncbi.nlm.nih.gov/protein/CDB93667.1>) |
|  | *Lachnospiraceae_bacterium* | 0.600 (0.030,1.170) |  | Sasaki K, Inoue J, Sasaki D, Hoshi N, Shirai T, Fukuda I, et al. Construction of a model culture system of human colonic microbiota to detect decreased *Lachnospiraceae* abundance and butyrogenesis in the feces of ulcerative colitis patients. Biotechnol J. 2019;14(5):e1800555. |
|  |  |  |  |  |
| **Cellobiose (β-1,4)** |  |  |  |  |
|  | *Faecalibacterium prausnitzii* | 0.645 (0.100,1.190) | Beta-glucosidase | GHJ81845.1  (National Center for Biotechnology Information. beta-glucosidase [*Faecalibacterium prausnitzii*] (GenBank: GHJ81845.1). Bethesda (MD): National Library of Medicine (US); 2023 [cited 2025 Dec 20]. Available from: <https://www.ncbi.nlm.nih.gov/protein/GHJ81845.1>) |
|  | *Megamonas funiformis* | 0.995 (0.460,1.530) | PTS lactose/cellobiose transporter subunit IIA | QIB59259.1  (National Center for Biotechnology Information. PTS lactose/cellobiose transporter subunit IIA [*Megamonas funiformis*] (GenBank: QIB59259.1). Bethesda (MD): National Library of Medicine (US); 2020 [cited 2025 Dec 20]. Available from: <https://www.ncbi.nlm.nih.gov/protein/QIB59259.1>) |
|  |  |  | 6-phospho-beta-glucosidase | QIB59260.1  (National Center for Biotechnology Information. 6-phospho-beta-glucosidase [*Megamonas funiformis*] (GenBank: QIB59260.1). Bethesda (MD): National Library of Medicine (US); 2020 [cited 2025 Dec 20]. Available from: <https://www.ncbi.nlm.nih.gov/protein/QIB59260.1>) |
|  | *Blautia obeum* | 0.775 (0.260,1.290) | Beta-galactosidase(with cross-activity) | CBL23046.1  (National Center for Biotechnology Information. beta-galactosidase [*Blautia obeum* A2-162] (GenBank: CBL23046.1). Bethesda (MD): National Library of Medicine (US); 2015 [cited 2025 Dec 20]. Available from: <https://www.ncbi.nlm.nih.gov/protein/CBL23046.1>) |
|  | *Faecalibacillus intestinalis* | 0.995 (0.470,1.520) | Hypothetical protein (putative glycosidase) | BCL58477.1  (National Center for Biotechnology Information. hypothetical protein Fi14EGH31_21890 [*Faecalibacillus intestinalis*] (GenBank: BCL58477.1). Bethesda (MD): National Library of Medicine (US); 2020 [cited 2025 Dec 20]. Available from: <https://www.ncbi.nlm.nih.gov/protein/BCL58477.1>) |
|  |  |  |  |  |
| **Gentiobiose (β-1,6)** |  |  |  |  |
|  | *Bifidobacterium pseudocatenulatum* | 0.655 (0.130,1.180) | Oligo-1,6-glucosidase | VUX65489.1  (National Center for Biotechnology Information. Oligo-1,6-glucosidase [*Bifidobacterium pseudocatenulatum*] (GenBank: VUX65489.1). Bethesda (MD): National Library of Medicine (US); 2019 [cited 2025 Dec 20]. Available from: <https://www.ncbi.nlm.nih.gov/protein/VUX65489.1>) |
|  | *Faecalibacillus intestinalis* | 0.540 (0.010,1.070) | Hypothetical protein (putative glycosidase) | BCL58477.1  (National Center for Biotechnology Information. hypothetical protein Fi14EGH31_21890 [*Faecalibacillus intestinalis*] (GenBank: BCL58477.1). Bethesda (MD): National Library of Medicine (US); 2020 [cited 2025 Dec 20]. Available from: <https://www.ncbi.nlm.nih.gov/protein/BCL58477.1>) |

**Table S7. Detailed information and statistical summary of differential metabolites between the Glucose fermentation group and the blank control.**

| **No.** | **Metabolites** | **Compound ID** | **Class** | **Sub Class** | **logFC** | **adj. *p*-value** | **Hotelling-T2** | **m/z** | **Retention time (min)** |
| --- | --- | --- | --- | --- | --- | --- | --- | --- | --- |
| MT01452 | N-((6-Methyl-5-oxo-3-thiomorpholinyl)carbonyl)-L-histidyl-L-proline | HMDB0250383 | Diazanaphthalenes | Benzodiazines | 1.0424 | 0.0032 | 511.9200 | 817.2727 | 7.0953 |
| MT02302 | Cytosine deoxyribonucleoside | HMDB0250725 | Pyrimidine nucleosides | Pyrimidine 2'-deoxyribonucleosides | 1.0243 | 0.0028 | 448.4900 | 272.0880 | 2.6198 |
| MT01107 | 3,5-O-METHYLENE-MESO-GLYCERO-GULO-HEPTITOL | 988786 | Unclassified | Unclassified | -1.3940 | 0.0000 | 655.3700 | 447.1708 | 4.7571 |

**Table S8. Detailed information and statistical summary of differential metabolites between the Trehalose fermentation group and the blank control.**

| **No.** | **Metabolites** | **Compound ID** | **Class** | **Sub Class** | **logFC** | **adj. *p*-value** | **Hotelling-T2** | **m/z** | **Retention time (min)** |
| --- | --- | --- | --- | --- | --- | --- | --- | --- | --- |
| MT00159 | Valyllysine | HMDB0029132 | Carboxylic acids and derivatives | Amino acids, peptides, and analogues | 1.0668 | 0.0009 | 880.5800 | 244.1659 | 6.4815 |
| MT00186 | S-Prenyl-L-cysteine | HMDB0012286 | Carboxylic acids and derivatives | Amino acids, peptides, and analogues | 1.4065 | 0.0000 | 1192.5000 | 190.0887 | 1.5974 |
| MT00193 | Asparaginyl-Proline | HMDB0028739 | Carboxylic acids and derivatives | Amino acids, peptides, and analogues | 1.2995 | 0.0001 | 1181.9000 | 210.0873 | 6.0150 |
| MT00248 | Threonylvaline | HMDB0029074 | Carboxylic acids and derivatives | Amino acids, peptides, and analogues | 1.2128 | 0.0002 | 863.2100 | 217.1183 | 5.8876 |
| MT00251 | Thr-Leu | HMDB0259044 | Carboxylic acids and derivatives | Amino acids, peptides, and analogues | 1.2453 | 0.0002 | 986.7000 | 231.1341 | 5.8706 |
| MT00595 | Methionyl-Threonine | HMDB0028983 | Carboxylic acids and derivatives | Amino acids, peptides, and analogues | 1.3843 | 0.0001 | 918.7300 | 249.0908 | 5.8706 |
| MT00679 | Histidylarginine | HMDB0028879 | Carboxylic acids and derivatives | Amino acids, peptides, and analogues | 1.0773 | 0.0002 | 823.3500 | 334.1594 | 5.9332 |
| MT02196 | Phenylalanylthreonine | HMDB0029005 | Carboxylic acids and derivatives | Amino acids, peptides, and analogues | 1.0276 | 0.0002 | 722.0800 | 265.1186 | 2.9215 |
| MT02449 | N-Acetyl-3-(nitrosulfanyl)-L-valine | HMDB0244752 | Carboxylic acids and derivatives | Amino acids, peptides, and analogues | 1.2044 | 0.0000 | 770.5400 | 235.0386 | 7.3715 |
| MT02617 | Ala-Hyp-Gly | HMDB0248094 | Carboxylic acids and derivatives | Amino acids, peptides, and analogues | 1.1199 | 0.0001 | 825.6600 | 258.1088 | 1.4761 |
| MT02679 | Threonylhistidine | HMDB0029063 | Carboxylic acids and derivatives | Amino acids, peptides, and analogues | 1.2836 | 0.0000 | 800.3000 | 257.1235 | 7.1183 |
| MT00756 | N-[4-[Acetyl(3-aminopropyl)amino]butyl]-N-(3-aminopropyl)acetamide | HMDB0260383 | Carboxylic acids and derivatives | Carboxylic acid derivatives | 1.2890 | 0.0000 | 952.2400 | 331.2343 | 6.6301 |
| MT01333 | Methylmalonic acid | 3712 | Carboxylic acids and derivatives | Dicarboxylic acids and derivatives | 1.4956 | 0.0000 | 1046.0000 | 136.0610 | 3.4292 |
| MT01910 | 4,5-Dihydro-2,5-dimethyl-4-oxo-3-furanyl butyrate | HMDB0303217 | Fatty Acyls | Fatty acid esters | 1.1522 | 0.0001 | 888.6400 | 237.0526 | 7.5155 |
| MT01488 | (2S,3R,4R,6R,7S,8R,9R)-1,2,3,4,6,7,8,9-Octahydroxydecan-5-one | HMDB0255321 | Fatty Acyls | Fatty alcohols | 1.4069 | 0.0000 | 903.5400 | 329.1092 | 1.3579 |
| MT02139 | PGP(18:3(9Z,12Z,15Z)/22:6(4Z,7Z,10Z,13Z,16Z,19Z)) | HMDB0013591 | Glycerophospholipids | Glycerophosphoglycerophosphates | 1.0810 | 0.0001 | 771.5900 | 877.4403 | 7.3291 |
| MT00677 | Methyl 3-hydroxybutyrate | HMDB0041603 | Hydroxy acids and derivatives | Beta hydroxy acids and derivatives | -1.1076 | 0.0001 | 836.9400 | 235.1191 | 5.9046 |
| MT02880 | 5-Hydroxysebacate | HMDB0029189 | Hydroxy acids and derivatives | Medium-chain hydroxy acids and derivatives | 1.2276 | 0.0001 | 787.7400 | 236.1482 | 8.3743 |
| MT01533 | 1-[3,4-Dihydroxy-5-(hydroxymethyl)oxolan-2-yl]-5-hydroxyimidazole-4-carboxamide | HMDB0255794 | Imidazole ribonucleosides and ribonucleotides | 1-ribosyl-imidazolecarboxamides | 1.1677 | 0.0001 | 838.5400 | 277.1144 | 6.6699 |
| MT02220 | 1-Hydroxy-3-methyl-9H-carbazole | HMDB0033487 | Indoles and derivatives | Carbazoles | 1.6295 | 0.0000 | 1062.7000 | 393.1626 | 6.6514 |
| MT03115 | Methyl 2-(1H-indole-3-carbonyl)thiazole-4-carboxylate | HMDB0246680 | Indoles and derivatives | Indolecarboxylic acids and derivatives | 1.6149 | 0.0000 | 1216.9000 | 331.0395 | 6.1721 |
| MT00970 | (S)-5-Amino-3-oxohexanoate | HMDB0012131 | Keto acids and derivatives | Medium-chain keto acids and derivatives | -1.1013 | 0.0002 | 876.3900 | 190.0720 | 5.9217 |
| MT00555 | Ciliatine | HMDB0011747 | Organic phosphonic acids and derivatives | Organic phosphonic acids | 1.5600 | 0.0000 | 854.3900 | 170.0225 | 3.4212 |
| MT01490 | 5-(Methylthio)-2,3-dioxopentyl phosphate | HMDB0059620 | Organic phosphoric acids and derivatives | Phosphate esters | 1.5594 | 0.0000 | 798.4600 | 287.0002 | 1.4958 |
| MT01966 | 2-hydroxy-5-(methylthio)-3-oxopent-1-enyl phosphate | HMDB0304070 | Organic phosphoric acids and derivatives | Phosphate esters | 1.3545 | 0.0000 | 766.3700 | 287.0002 | 1.8312 |
| MT00680 | 2-Hydroxyisobutyrate | HMDB0242161 | Organooxygen compounds | Alcohols and polyols | -1.0241 | 0.0005 | 802.8300 | 205.0720 | 6.0150 |
| MT00076 | Galabiose | HMDB0029902 | Organooxygen compounds | Carbohydrates and carbohydrate conjugates | 1.1681 | 0.0000 | 971.4400 | 365.1039 | 3.7038 |
| MT00332 | Lacdinac | HMDB0253938 | Organooxygen compounds | Carbohydrates and carbohydrate conjugates | 1.1517 | 0.0008 | 809.0100 | 469.1658 | 6.4388 |
| MT00450 | 2,3,4-Trihydroxybutanoic acid | HMDB0245425 | Organooxygen compounds | Carbohydrates and carbohydrate conjugates | 1.4400 | 0.0000 | 1110.5000 | 135.0297 | 2.0977 |
| MT01173 | N-(1-Deoxy-1-fructosyl)alanine | HMDB0038662 | Organooxygen compounds | Carbohydrates and carbohydrate conjugates | 1.2335 | 0.0000 | 814.3400 | 296.0992 | 2.4317 |
| MT02622 | Creatine riboside | HMDB0240254 | Organooxygen compounds | Carbohydrates and carbohydrate conjugates | 1.1618 | 0.0001 | 853.0200 | 244.0930 | 1.5935 |
| MT00025 | 4-Hydroxybenzaldehyde | 62451 | Organooxygen compounds | Carbonyl compounds | 1.1500 | 0.0002 | 949.8100 | 123.0435 | 5.9332 |
| MT03230 | 3-Methoxy-4-Hydroxyphenylglycol sulfate | HMDB0003332 | Phenols | Methoxyphenols | 1.1247 | 0.0001 | 831.2100 | 245.0116 | 1.1881 |
| MT02412 | 7,8-dihydro-L-Biopterin | 5106 | Pteridines and derivatives | Pterins and derivatives | 1.1664 | 0.0001 | 875.3800 | 284.0993 | 4.5153 |
| MT02434 | 7,8-Dihydroneopterin | 3386 | Pteridines and derivatives | Pterins and derivatives | 1.1279 | 0.0002 | 864.6900 | 254.0888 | 6.4388 |
| MT00307 | cAMP | 92 | Purine nucleotides | Cyclic purine nucleotides | 1.3473 | 0.0000 | 855.6600 | 328.0444 | 9.9324 |
| MT01302 | DG(8:0/0:0/20:5(7Z,9Z,11E,13E,17Z)-3OH(5,6,15)) | HMDB0297205 | Unclassified | Unclassified | -1.4345 | 0.0000 | 773.0400 | 595.3483 | 0.9538 |
| MT02270 | PG(18:1(9Z)-O(12,13)/18:0) | HMDB0268822 | Unclassified | Unclassified | 1.2182 | 0.0001 | 929.0200 | 835.5315 | 1.0819 |

**Table S9. Detailed information and statistical summary of differential metabolites between the Maltose fermentation group and the blank control.**

| **No.** | **Metabolites** | **Compound ID** | **Class** | **Sub Class** | **logFC** | **adj. *p*-value** | **Hotelling-T2** | **m/z** | **Retention time (min)** |
| --- | --- | --- | --- | --- | --- | --- | --- | --- | --- |
| MT01259 | 5-Hydroxy-1-methylhydantoin | HMDB0246800 | Azolidines | Imidazolidines | 1.3310 | 0.0000 | 1316.5000 | 259.0692 | 5.8706 |
| MT00044 | Saccharopine | 383 | Carboxylic acids and derivatives | Amino acids, peptides, and analogues | 1.2795 | 0.0003 | 1212.0000 | 275.1243 | 9.6176 |
| MT00138 | 2-Hydroxy-L-methionine | HMDB0246915 | Carboxylic acids and derivatives | Amino acids, peptides, and analogues | 1.5028 | 0.0000 | 1387.0000 | 148.0420 | 2.1777 |
| MT00172 | Phenylalanylhydroxyproline | HMDB0011176 | Carboxylic acids and derivatives | Amino acids, peptides, and analogues | 1.2370 | 0.0005 | 1064.3000 | 277.1186 | 5.9046 |
| MT00179 | Hydroxyprolyl-Asparagine | HMDB0028858 | Carboxylic acids and derivatives | Amino acids, peptides, and analogues | 1.2589 | 0.0004 | 965.0200 | 226.0824 | 6.0353 |
| MT00186 | S-Prenyl-L-cysteine | HMDB0012286 | Carboxylic acids and derivatives | Amino acids, peptides, and analogues | 1.5006 | 0.0000 | 1388.4000 | 190.0887 | 1.5974 |
| MT00226 | Serylhydroxyproline | HMDB0029040 | Carboxylic acids and derivatives | Amino acids, peptides, and analogues | 1.1791 | 0.0017 | 985.1700 | 217.0820 | 5.8876 |
| MT00391 | L-Cystine | HMDB0000192 | Carboxylic acids and derivatives | Amino acids, peptides, and analogues | 1.2949 | 0.0001 | 951.8900 | 239.0157 | 9.7864 |
| MT00679 | Histidylarginine | HMDB0028879 | Carboxylic acids and derivatives | Amino acids, peptides, and analogues | 1.0827 | 0.0004 | 884.4600 | 334.1594 | 5.9332 |
| MT00851 | Histidine glutamate | HMDB0253183 | Carboxylic acids and derivatives | Amino acids, peptides, and analogues | 1.1166 | 0.0036 | 935.5100 | 345.1058 | 5.8876 |
| MT01146 | L-Aspartate-semialdehyde | HMDB0012249 | Carboxylic acids and derivatives | Amino acids, peptides, and analogues | 1.5291 | 0.0000 | 1416.0000 | 118.0496 | 1.1525 |
| MT01444 | 2-[(2S)-2-Amino-3-carboxypropanoyl]oxybutanedioic acid | HMDB0258136 | Carboxylic acids and derivatives | Amino acids, peptides, and analogues | 1.4073 | 0.0001 | 1298.6000 | 267.0816 | 6.4989 |
| MT02454 | Asparaginyl-Threonine | HMDB0028741 | Carboxylic acids and derivatives | Amino acids, peptides, and analogues | 1.3390 | 0.0000 | 1234.3000 | 251.1359 | 7.8763 |
| MT02530 | Ethiin | HMDB0037057 | Carboxylic acids and derivatives | Amino acids, peptides, and analogues | 1.4956 | 0.0000 | 1372.9000 | 148.0420 | 3.3606 |
| MT02547 | Threoninyl-Aspartate | HMDB0304789 | Carboxylic acids and derivatives | Amino acids, peptides, and analogues | 1.4597 | 0.0000 | 1347.3000 | 235.0914 | 5.9161 |
| MT02617 | Ala-Hyp-Gly | HMDB0248094 | Carboxylic acids and derivatives | Amino acids, peptides, and analogues | 1.1096 | 0.0003 | 879.3200 | 258.1088 | 1.4761 |
| MT00756 | N-[4-[Acetyl(3-aminopropyl)amino]butyl]-N-(3-aminopropyl)acetamide | HMDB0260383 | Carboxylic acids and derivatives | Carboxylic acid derivatives | 1.0830 | 0.0015 | 941.4400 | 331.2343 | 6.6301 |
| MT00846 | Iminoaspartic acid | HMDB0001131 | Carboxylic acids and derivatives | Dicarboxylic acids and derivatives | 1.5117 | 0.0000 | 1425.1000 | 169.9850 | 4.3562 |
| MT00828 | Sodium citrate | HMDB0303752 | Carboxylic acids and derivatives | Tricarboxylic acids and derivatives | -1.5212 | 0.0000 | 1534.2000 | 259.9884 | 1.3380 |
| MT01416 | 2-Isopropyl citrate | HMDB0038083 | Carboxylic acids and derivatives | Tricarboxylic acids and derivatives | 1.6069 | 0.0000 | 1088.2000 | 252.1077 | 2.9808 |
| MT02737 | 3,5-Dihydroxycinnamic acid sulfate | HMDB0240449 | Cinnamic acids and derivatives | Hydroxycinnamic acids and derivatives | 1.3810 | 0.0000 | 1104.7000 | 518.9920 | 2.3262 |
| MT00835 | Indolo[2,1-b]quinazoline-6,12-dione | HMDB0253473 | Diazanaphthalenes | Benzodiazines | 1.5265 | 0.0000 | 1292.7000 | 495.1107 | 11.7079 |
| MT01910 | 4,5-Dihydro-2,5-dimethyl-4-oxo-3-furanyl butyrate | HMDB0303217 | Fatty Acyls | Fatty acid esters | 1.1402 | 0.0002 | 935.0700 | 237.0526 | 7.5155 |
| MT00852 | Succinamic acid | HMDB0258538 | Fatty Acyls | Fatty acids and conjugates | 1.0484 | 0.0044 | 855.9400 | 233.0770 | 5.9046 |
| MT02092 | 3-S-methylmercaptopropionate | HMDB0304155 | Fatty Acyls | Fatty acids and conjugates | 1.3599 | 0.0000 | 1009.2000 | 137.0509 | 2.8100 |
| MT01488 | (2S,3R,4R,6R,7S,8R,9R)-1,2,3,4,6,7,8,9-Octahydroxydecan-5-one | HMDB0255321 | Fatty Acyls | Fatty alcohols | 1.3219 | 0.0001 | 1257.6000 | 329.1092 | 1.3579 |
| MT03069 | PC(20:4(8Z,11Z,14Z,17Z)/0:0) | LMGP01050140 | Glycerophospholipids | Glycerophosphocholines | 1.3814 | 0.0000 | 902.5800 | 582.2980 | 1.2897 |
| MT02808 | LysoPE(0:0/15:0) | HMDB0011472 | Glycerophospholipids | Glycerophosphoethanolamines | 1.4246 | 0.0000 | 1441.4000 | 438.2647 | 1.3380 |
| MT00164 | Glycerophosphoinositol | HMDB0011649 | Glycerophospholipids | Glycerophosphoinositols | 1.4729 | 0.0000 | 978.1100 | 333.0582 | 11.0293 |
| MT01267 | 6,8-Dihydroxypurine | HMDB0001182 | Imidazopyrimidines | Purines and purine derivatives | 1.2543 | 0.0006 | 1376.1000 | 303.0591 | 6.1121 |
| MT01751 | 2-Hydroxypurine | HMDB0245165 | Imidazopyrimidines | Purines and purine derivatives | 1.5277 | 0.0000 | 1403.1000 | 137.0452 | 1.8190 |
| MT01776 | (6-Aminopurin-9-yl) [hydroxy(phosphonooxy)phosphoryl] hydrogen phosphate | HMDB0258010 | Imidazopyrimidines | Purines and purine derivatives | 1.4919 | 0.0000 | 1425.0000 | 373.9459 | 4.3734 |
| MT02912 | 2',3'-Dialdehyde ATP | HMDB0255984 | Imidazopyrimidines | Purines and purine derivatives | 1.5104 | 0.0000 | 1377.4000 | 485.9642 | 2.1787 |
| MT03115 | Methyl 2-(1H-indole-3-carbonyl)thiazole-4-carboxylate | HMDB0246680 | Indoles and derivatives | Indolecarboxylic acids and derivatives | 1.8267 | 0.0000 | 1293.6000 | 331.0395 | 6.1721 |
| MT01200 | Dihydroxyfumaric acid | HMDB0002050 | Keto acids and derivatives | Short-chain keto acids and derivatives | 1.5258 | 0.0000 | 1374.4000 | 294.9950 | 6.8628 |
| MT00638 | 2-Carboxyoxypropanoic acid | HMDB0257720 | Organic carbonic acids and derivatives | Carbonic acid monoesters | 1.4890 | 0.0000 | 1343.2000 | 152.0558 | 2.2974 |
| MT01417 | DL-Glyceraldehyde 2-Phosphate | 103468 | Organic phosphoric acids and derivatives | Phosphate esters | 1.3504 | 0.0001 | 1369.1000 | 338.9903 | 4.3492 |
| MT01385 | 2,2'-Dithiodiethanesulfonic acid | HMDB0246681 | Organic sulfonic acids and derivatives | Organosulfonic acids and derivatives | -1.5225 | 0.0000 | 1527.4000 | 262.9173 | 1.0447 |
| MT02984 | o-Cresol sulfate | HMDB0240655 | Organic sulfuric acids and derivatives | Arylsulfates | 1.4817 | 0.0000 | 1355.7000 | 206.0472 | 1.7338 |
| MT03145 | 3,5-Dihydroxybenzoic acid sulfate | HMDB0240373 | Organic sulfuric acids and derivatives | Arylsulfates | -1.8552 | 0.0000 | 955.2000 | 232.9751 | 1.0819 |
| MT02332 | 6-amino-5[N-methylformylamino]-1-methyluracil | HMDB0059771 | Organonitrogen compounds | N-arylamides | 1.3553 | 0.0000 | 1215.2000 | 199.0817 | 6.0702 |
| MT00291 | Dolichyl phosphate D-mannose | HMDB0000994 | Organooxygen compounds | Carbohydrates and carbohydrate conjugates | 1.1990 | 0.0001 | 928.1300 | 241.0114 | 9.7864 |
| MT01173 | N-(1-Deoxy-1-fructosyl)alanine | HMDB0038662 | Organooxygen compounds | Carbohydrates and carbohydrate conjugates | 1.3206 | 0.0000 | 968.5700 | 296.0992 | 2.4317 |
| MT01849 | D-Xylose, 4-O-beta-D-xylopyranosyl- | HMDB0250808 | Organooxygen compounds | Carbohydrates and carbohydrate conjugates | 1.4022 | 0.0000 | 894.4200 | 305.0839 | 1.8361 |
| MT02198 | 5-phospho-alpha-D-ribose 1-diphosphate | HMDB0304220 | Organooxygen compounds | Carbohydrates and carbohydrate conjugates | 1.3791 | 0.0000 | 1387.0000 | 383.9035 | 3.9060 |
| MT02948 | Glucocapparin | HMDB0304680 | Organooxygen compounds | Carbohydrates and carbohydrate conjugates | 1.2039 | 0.0004 | 1218.7000 | 331.0033 | 7.0317 |
| MT00025 | 4-Hydroxybenzaldehyde | 62451 | Organooxygen compounds | Carbonyl compounds | 1.1731 | 0.0002 | 1019.2000 | 123.0435 | 5.9332 |
| MT03230 | 3-Methoxy-4-Hydroxyphenylglycol sulfate | HMDB0003332 | Phenols | Methoxyphenols | 1.2016 | 0.0001 | 965.8100 | 245.0116 | 1.1881 |
| MT02412 | 7,8-dihydro-L-Biopterin | 5106 | Pteridines and derivatives | Pterins and derivatives | 1.1713 | 0.0002 | 952.2000 | 284.0993 | 4.5153 |
| MT00307 | cAMP | 92 | Purine nucleotides | Cyclic purine nucleotides | 1.0256 | 0.0009 | 972.0900 | 328.0444 | 9.9324 |
| MT03210 | Adenosine phosphosulfate | HMDB0001003 | Purine nucleotides | Purine ribonucleotides | 1.4641 | 0.0000 | 1303.4000 | 426.0114 | 7.9864 |
| MT02364 | dUDP | HMDB0001000 | Pyrimidine nucleotides | Pyrimidine deoxyribonucleotides | 1.4957 | 0.0000 | 1444.0000 | 387.0016 | 9.0661 |
| MT03372 | Cholylarginine | HMDB0242375 | Steroids and steroid derivatives | Bile acids, alcohols and derivatives | 1.3631 | 0.0000 | 1197.7000 | 565.3933 | 5.9846 |
| MT01775 | 3h-Taurine | HMDB0246277 | Thiocarbonyl compounds | Thioaldehydes | 1.5074 | 0.0000 | 1408.4000 | 126.0215 | 4.3562 |
| MT01078 | S-2-Propenyl propanethioate | HMDB0037493 | Thiocarboxylic acids and derivatives | Thioesters | 1.5211 | 0.0000 | 1401.1000 | 131.0526 | 1.2555 |
| MT02810 | 2-Thioxothiazolidine-4-carboxylic acid | HMDB0301802 | Thiolanes | Thiolane-2-thiones | -1.7315 | 0.0000 | 1490.5000 | 142.9628 | 1.4958 |
| MT00014 | (R)-(+)-2-Pyrrolidone-5-carboxylic acid | 63632 | Unclassified | Unclassified | 1.5022 | 0.0000 | 1143.7000 | 130.0493 | 1.2555 |
| MT01178 | 2-Iodophenol | 65668 | Unclassified | Unclassified | 1.5024 | 0.0000 | 1420.9000 | 237.9720 | 4.3734 |

**Table S10. Detailed information and statistical summary of differential metabolites between the Isomaltose fermentation group and the blank control.**

| **No.** | **Metabolites** | **Compound ID** | **Class** | **Sub Class** | **logFC** | **adj. *p*-value** | **Hotelling-T2** | **m/z** | **Retention time (min)** |
| --- | --- | --- | --- | --- | --- | --- | --- | --- | --- |
| MT00004 | L-Proline | 29 | Carboxylic acids and derivatives | Amino acids, peptides, and analogues | 1.4238 | 0.0000 | 928.6700 | 116.0704 | 6.3958 |
| MT00039 | Trans-4-Hydroxy-L-proline | 257 | Carboxylic acids and derivatives | Amino acids, peptides, and analogues | 1.5224 | 0.0000 | 831.2100 | 132.0649 | 6.7381 |
| MT00044 | Saccharopine | 383 | Carboxylic acids and derivatives | Amino acids, peptides, and analogues | 1.3511 | 0.0001 | 972.8000 | 275.1243 | 9.6176 |
| MT00138 | 2-Hydroxy-L-methionine | HMDB0246915 | Carboxylic acids and derivatives | Amino acids, peptides, and analogues | 1.3596 | 0.0000 | 1003.9000 | 148.0420 | 2.1777 |
| MT00172 | Phenylalanylhydroxyproline | HMDB0011176 | Carboxylic acids and derivatives | Amino acids, peptides, and analogues | 1.4370 | 0.0000 | 881.2100 | 277.1186 | 5.9046 |
| MT00179 | Hydroxyprolyl-Asparagine | HMDB0028858 | Carboxylic acids and derivatives | Amino acids, peptides, and analogues | 1.4140 | 0.0000 | 990.0600 | 226.0824 | 6.0353 |
| MT00186 | S-Prenyl-L-cysteine | HMDB0012286 | Carboxylic acids and derivatives | Amino acids, peptides, and analogues | 1.4952 | 0.0000 | 1258.9000 | 190.0887 | 1.5974 |
| MT00193 | Asparaginyl-Proline | HMDB0028739 | Carboxylic acids and derivatives | Amino acids, peptides, and analogues | 1.2576 | 0.0003 | 982.8700 | 210.0873 | 6.0150 |
| MT01146 | L-Aspartate-semialdehyde | HMDB0012249 | Carboxylic acids and derivatives | Amino acids, peptides, and analogues | 1.5327 | 0.0000 | 1366.4000 | 118.0496 | 1.1525 |
| MT01983 | N-Salicyloylaspartic acid | HMDB0039506 | Carboxylic acids and derivatives | Amino acids, peptides, and analogues | 1.2761 | 0.0001 | 873.3200 | 505.1107 | 3.6203 |
| MT02150 | Prolylproline | HMDB0011180 | Carboxylic acids and derivatives | Amino acids, peptides, and analogues | 1.2743 | 0.0003 | 813.7400 | 257.1136 | 9.5964 |
| MT02449 | N-Acetyl-3-(nitrosulfanyl)-L-valine | HMDB0244752 | Carboxylic acids and derivatives | Amino acids, peptides, and analogues | 1.1993 | 0.0001 | 845.1500 | 235.0386 | 7.3715 |
| MT02454 | Asparaginyl-Threonine | HMDB0028741 | Carboxylic acids and derivatives | Amino acids, peptides, and analogues | 1.4788 | 0.0000 | 1332.1000 | 251.1359 | 7.8763 |
| MT02530 | Ethiin | HMDB0037057 | Carboxylic acids and derivatives | Amino acids, peptides, and analogues | 1.3511 | 0.0000 | 989.5300 | 148.0420 | 3.3606 |
| MT02547 | Threoninyl-Aspartate | HMDB0304789 | Carboxylic acids and derivatives | Amino acids, peptides, and analogues | 1.4636 | 0.0000 | 1237.8000 | 235.0914 | 5.9161 |
| MT02617 | Ala-Hyp-Gly | HMDB0248094 | Carboxylic acids and derivatives | Amino acids, peptides, and analogues | 1.1128 | 0.0001 | 830.8600 | 258.1088 | 1.4761 |
| MT01416 | 2-Isopropyl citrate | HMDB0038083 | Carboxylic acids and derivatives | Tricarboxylic acids and derivatives | 1.6095 | 0.0000 | 980.4900 | 252.1077 | 2.9808 |
| MT01910 | 4,5-Dihydro-2,5-dimethyl-4-oxo-3-furanyl butyrate | HMDB0303217 | Fatty Acyls | Fatty acid esters | 1.1300 | 0.0002 | 918.7400 | 237.0526 | 7.5155 |
| MT02092 | 3-S-methylmercaptopropionate | HMDB0304155 | Fatty Acyls | Fatty acids and conjugates | 1.5072 | 0.0000 | 1314.5000 | 137.0509 | 2.8100 |
| MT01488 | (2S,3R,4R,6R,7S,8R,9R)-1,2,3,4,6,7,8,9-Octahydroxydecan-5-one | HMDB0255321 | Fatty Acyls | Fatty alcohols | 1.4704 | 0.0000 | 1062.0000 | 329.1092 | 1.3579 |
| MT03069 | PC(20:4(8Z,11Z,14Z,17Z)/0:0) | LMGP01050140 | Glycerophospholipids | Glycerophosphocholines | 1.3888 | 0.0000 | 1093.3000 | 582.2980 | 1.2897 |
| MT02808 | LysoPE(0:0/15:0) | HMDB0011472 | Glycerophospholipids | Glycerophosphoethanolamines | 1.2670 | 0.0001 | 1146.5000 | 438.2647 | 1.3380 |
| MT01257 | Formyl 3-hydroxybutanoate | HMDB0257659 | Hydroxy acids and derivatives | Beta hydroxy acids and derivatives | 1.2652 | 0.0000 | 902.1200 | 150.0764 | 3.5492 |
| MT01267 | 6,8-Dihydroxypurine | HMDB0001182 | Imidazopyrimidines | Purines and purine derivatives | 1.2486 | 0.0004 | 1323.6000 | 303.0591 | 6.1121 |
| MT01751 | 2-Hydroxypurine | HMDB0245165 | Imidazopyrimidines | Purines and purine derivatives | 1.5276 | 0.0000 | 1335.3000 | 137.0452 | 1.8190 |
| MT01776 | (6-Aminopurin-9-yl) [hydroxy(phosphonooxy)phosphoryl] hydrogen phosphate | HMDB0258010 | Imidazopyrimidines | Purines and purine derivatives | 1.3577 | 0.0000 | 1348.0000 | 373.9459 | 4.3734 |
| MT02912 | 2',3'-Dialdehyde ATP | HMDB0255984 | Imidazopyrimidines | Purines and purine derivatives | 1.3664 | 0.0000 | 966.2800 | 485.9642 | 2.1787 |
| MT03115 | Methyl 2-(1H-indole-3-carbonyl)thiazole-4-carboxylate | HMDB0246680 | Indoles and derivatives | Indolecarboxylic acids and derivatives | 1.7347 | 0.0000 | 862.1000 | 331.0395 | 6.1721 |
| MT01200 | Dihydroxyfumaric acid | HMDB0002050 | Keto acids and derivatives | Short-chain keto acids and derivatives | 1.4278 | 0.0000 | 1094.6000 | 294.9950 | 6.8628 |
| MT00638 | 2-Carboxyoxypropanoic acid | HMDB0257720 | Organic carbonic acids and derivatives | Carbonic acid monoesters | 1.4641 | 0.0000 | 1088.9000 | 152.0558 | 2.2974 |
| MT01417 | DL-Glyceraldehyde 2-Phosphate | 103468 | Organic phosphoric acids and derivatives | Phosphate esters | 1.4913 | 0.0000 | 1352.1000 | 338.9903 | 4.3492 |
| MT02984 | o-Cresol sulfate | HMDB0240655 | Organic sulfuric acids and derivatives | Arylsulfates | 1.3400 | 0.0000 | 993.2100 | 206.0472 | 1.7338 |
| MT03145 | 3,5-Dihydroxybenzoic acid sulfate | HMDB0240373 | Organic sulfuric acids and derivatives | Arylsulfates | -1.5756 | 0.0000 | 904.5500 | 232.9751 | 1.0819 |
| MT00155 | (E)-2-Penten-1-ol | HMDB0031604 | Organooxygen compounds | Alcohols and polyols | 1.1996 | 0.0001 | 894.7800 | 104.1070 | 3.1544 |
| MT00208 | D-Glucamine | HMDB0246693 | Organooxygen compounds | Carbohydrates and carbohydrate conjugates | 1.3865 | 0.0000 | 813.6500 | 164.0921 | 1.6316 |
| MT00332 | Lacdinac | HMDB0253938 | Organooxygen compounds | Carbohydrates and carbohydrate conjugates | 1.4357 | 0.0000 | 911.1100 | 469.1658 | 6.4388 |
| MT00450 | 2,3,4-Trihydroxybutanoic acid | HMDB0245425 | Organooxygen compounds | Carbohydrates and carbohydrate conjugates | 1.5625 | 0.0000 | 1234.4000 | 135.0297 | 2.0977 |
| MT01043 | L-Arabitol | HMDB0001851 | Organooxygen compounds | Carbohydrates and carbohydrate conjugates | 1.2912 | 0.0000 | 837.8200 | 197.0668 | 6.0539 |
| MT01173 | N-(1-Deoxy-1-fructosyl)alanine | HMDB0038662 | Organooxygen compounds | Carbohydrates and carbohydrate conjugates | 1.3182 | 0.0000 | 862.6200 | 296.0992 | 2.4317 |
| MT01849 | D-Xylose, 4-O-beta-D-xylopyranosyl- | HMDB0250808 | Organooxygen compounds | Carbohydrates and carbohydrate conjugates | 1.3981 | 0.0000 | 1104.9000 | 305.0839 | 1.8361 |
| MT02198 | 5-phospho-alpha-D-ribose 1-diphosphate | HMDB0304220 | Organooxygen compounds | Carbohydrates and carbohydrate conjugates | 1.3776 | 0.0000 | 1031.8000 | 383.9035 | 3.9060 |
| MT02622 | Creatine riboside | HMDB0240254 | Organooxygen compounds | Carbohydrates and carbohydrate conjugates | 1.1777 | 0.0000 | 851.5200 | 244.0930 | 1.5935 |
| MT00025 | 4-Hydroxybenzaldehyde | 62451 | Organooxygen compounds | Carbonyl compounds | 1.1782 | 0.0001 | 965.6000 | 123.0435 | 5.9332 |
| MT03230 | 3-Methoxy-4-Hydroxyphenylglycol sulfate | HMDB0003332 | Phenols | Methoxyphenols | 1.2110 | 0.0000 | 898.7000 | 245.0116 | 1.1881 |
| MT02412 | 7,8-dihydro-L-Biopterin | 5106 | Pteridines and derivatives | Pterins and derivatives | 1.1917 | 0.0000 | 892.0200 | 284.0993 | 4.5153 |
| MT02434 | 7,8-Dihydroneopterin | 3386 | Pteridines and derivatives | Pterins and derivatives | 1.1328 | 0.0001 | 865.3900 | 254.0888 | 6.4388 |
| MT03210 | Adenosine phosphosulfate | HMDB0001003 | Purine nucleotides | Purine ribonucleotides | 1.4704 | 0.0000 | 1197.7000 | 426.0114 | 7.9864 |
| MT02238 | 4-Pyridylethylmercaptan | HMDB0246578 | Pyridines and derivatives | Unclassified | 1.3170 | 0.0000 | 994.8700 | 157.0796 | 7.9451 |
| MT02364 | dUDP | HMDB0001000 | Pyrimidine nucleotides | Pyrimidine deoxyribonucleotides | 1.3531 | 0.0000 | 1046.0000 | 387.0016 | 9.0661 |
| MT01775 | 3h-Taurine | HMDB0246277 | Thiocarbonyl compounds | Thioaldehydes | 1.5075 | 0.0000 | 1341.2000 | 126.0215 | 4.3562 |
| MT01078 | S-2-Propenyl propanethioate | HMDB0037493 | Thiocarboxylic acids and derivatives | Thioesters | 1.5204 | 0.0000 | 1333.2000 | 131.0526 | 1.2555 |
| MT00008 | AMINOHYDROXYBUTYRIC ACID | 44197 | Unclassified | Unclassified | 1.3909 | 0.0000 | 925.0600 | 120.0652 | 6.1043 |
| MT00014 | (R)-(+)-2-Pyrrolidone-5-carboxylic acid | 63632 | Unclassified | Unclassified | 1.4971 | 0.0000 | 1139.0000 | 130.0493 | 1.2555 |

**Table S11. Detailed information and statistical summary of differential metabolites between the Cellobiose fermentation group and the blank control.**

| **No.** | **Metabolites** | **Compound ID** | **Class** | **Sub Class** | **logFC** | **adj. *p*-value** | **Hotelling-T2** | **m/z** | **Retention time (min)** |
| --- | --- | --- | --- | --- | --- | --- | --- | --- | --- |
| MT01259 | 5-Hydroxy-1-methylhydantoin | HMDB0246800 | Azolidines | Imidazolidines | 1.3315 | 0.0000 | 1202.8000 | 259.0692 | 5.8706 |
| MT00186 | S-Prenyl-L-cysteine | HMDB0012286 | Carboxylic acids and derivatives | Amino acids, peptides, and analogues | 1.0398 | 0.0001 | 1006.5000 | 190.0887 | 1.5974 |
| MT00193 | Asparaginyl-Proline | HMDB0028739 | Carboxylic acids and derivatives | Amino acids, peptides, and analogues | 1.1565 | 0.0012 | 903.1800 | 210.0873 | 6.0150 |
| MT00595 | Methionyl-Threonine | HMDB0028983 | Carboxylic acids and derivatives | Amino acids, peptides, and analogues | 1.4583 | 0.0000 | 658.1600 | 249.0908 | 5.8706 |
| MT01983 | N-Salicyloylaspartic acid | HMDB0039506 | Carboxylic acids and derivatives | Amino acids, peptides, and analogues | 1.1578 | 0.0007 | 782.5100 | 505.1107 | 3.6203 |
| MT02055 | (4R)-3-((2S)-3-Mercapto-2-methylpropanoyl)-4-thiazolidinecarboxylic acid | HMDB0259957 | Carboxylic acids and derivatives | Amino acids, peptides, and analogues | 1.0439 | 0.0001 | 1090.9000 | 280.0310 | 1.0639 |
| MT02449 | N-Acetyl-3-(nitrosulfanyl)-L-valine | HMDB0244752 | Carboxylic acids and derivatives | Amino acids, peptides, and analogues | 1.1536 | 0.0004 | 818.9700 | 235.0386 | 7.3715 |
| MT02453 | Histidylserine | HMDB0028894 | Carboxylic acids and derivatives | Amino acids, peptides, and analogues | 1.1783 | 0.0001 | 664.5700 | 243.1080 | 7.6707 |
| MT02617 | Ala-Hyp-Gly | HMDB0248094 | Carboxylic acids and derivatives | Amino acids, peptides, and analogues | 1.0992 | 0.0002 | 783.1500 | 258.1088 | 1.4761 |
| MT02679 | Threonylhistidine | HMDB0029063 | Carboxylic acids and derivatives | Amino acids, peptides, and analogues | 1.2793 | 0.0000 | 682.3800 | 257.1235 | 7.1183 |
| MT00756 | N-[4-[Acetyl(3-aminopropyl)amino]butyl]-N-(3-aminopropyl)acetamide | HMDB0260383 | Carboxylic acids and derivatives | Carboxylic acid derivatives | 1.2657 | 0.0000 | 897.0600 | 331.2343 | 6.6301 |
| MT00835 | Indolo[2,1-b]quinazoline-6,12-dione | HMDB0253473 | Diazanaphthalenes | Benzodiazines | 1.1280 | 0.0002 | 790.7100 | 495.1107 | 11.7079 |
| MT01910 | 4,5-Dihydro-2,5-dimethyl-4-oxo-3-furanyl butyrate | HMDB0303217 | Fatty Acyls | Fatty acid esters | 1.1158 | 0.0004 | 872.8100 | 237.0526 | 7.5155 |
| MT02424 | 2-Ethyl-2-hydroxybutanedioylcarnitine | HMDB0241794 | Fatty Acyls | Fatty acid esters | 1.3316 | 0.0000 | 1131.0000 | 323.1813 | 6.0702 |
| MT02092 | 3-S-methylmercaptopropionate | HMDB0304155 | Fatty Acyls | Fatty acids and conjugates | 1.0791 | 0.0003 | 775.8900 | 137.0509 | 2.8100 |
| MT01257 | Formyl 3-hydroxybutanoate | HMDB0257659 | Hydroxy acids and derivatives | Beta hydroxy acids and derivatives | 1.2567 | 0.0000 | 1017.1000 | 150.0764 | 3.5492 |
| MT02554 | 3-Methyl-1-propylxanthine | HMDB0245925 | Imidazopyrimidines | Purines and purine derivatives | 1.1267 | 0.0008 | 902.2100 | 253.0934 | 6.0353 |
| MT02220 | 1-Hydroxy-3-methyl-9H-carbazole | HMDB0033487 | Indoles and derivatives | Carbazoles | 1.4925 | 0.0000 | 749.8500 | 393.1626 | 6.6514 |
| MT03115 | Methyl 2-(1H-indole-3-carbonyl)thiazole-4-carboxylate | HMDB0246680 | Indoles and derivatives | Indolecarboxylic acids and derivatives | 1.3293 | 0.0000 | 1254.1000 | 331.0395 | 6.1721 |
| MT00431 | TETRATHIONIC ACID | HMDB0258934 | Non-metal oxoanionic compounds | Non-metal tetrathionates | -1.3767 | 0.0000 | 790.6300 | 224.8651 | 2.3262 |
| MT01385 | 2,2'-Dithiodiethanesulfonic acid | HMDB0246681 | Organic sulfonic acids and derivatives | Organosulfonic acids and derivatives | -1.0751 | 0.0000 | 906.9900 | 262.9173 | 1.0447 |
| MT02009 | 2-Methylprop-2-ene-1-sulfonic acid | HMDB0245236 | Organic sulfonic acids and derivatives | Organosulfonic acids and derivatives | 1.2341 | 0.0001 | 775.9900 | 119.0157 | 6.1214 |
| MT02332 | 6-amino-5[N-methylformylamino]-1-methyluracil | HMDB0059771 | Organonitrogen compounds | N-arylamides | 1.4653 | 0.0000 | 1330.3000 | 199.0817 | 6.0702 |
| MT00492 | beta-tyvelose | HMDB0249152 | Organooxygen compounds | Carbohydrates and carbohydrate conjugates | 1.4751 | 0.0000 | 762.4300 | 193.0719 | 5.9945 |
| MT00833 | Phenylglucuronide | HMDB0059806 | Organooxygen compounds | Carbohydrates and carbohydrate conjugates | 1.2503 | 0.0000 | 866.8600 | 539.1394 | 10.8807 |
| MT01043 | L-Arabitol | HMDB0001851 | Organooxygen compounds | Carbohydrates and carbohydrate conjugates | 1.2555 | 0.0000 | 754.4800 | 197.0668 | 6.0539 |
| MT02506 | 2-O-Benzoyl-D-glucose | HMDB0034618 | Organooxygen compounds | Carbohydrates and carbohydrate conjugates | 1.1132 | 0.0001 | 1007.3000 | 307.0784 | 11.1624 |
| MT00025 | 4-Hydroxybenzaldehyde | 62451 | Organooxygen compounds | Carbonyl compounds | 1.1639 | 0.0002 | 926.0600 | 123.0435 | 5.9332 |
| MT03230 | 3-Methoxy-4-Hydroxyphenylglycol sulfate | HMDB0003332 | Phenols | Methoxyphenols | 1.1541 | 0.0001 | 803.4600 | 245.0116 | 1.1881 |
| MT02412 | 7,8-dihydro-L-Biopterin | 5106 | Pteridines and derivatives | Pterins and derivatives | 1.1908 | 0.0001 | 827.2800 | 284.0993 | 4.5153 |
| MT02434 | 7,8-Dihydroneopterin | 3386 | Pteridines and derivatives | Pterins and derivatives | 1.1297 | 0.0002 | 813.0300 | 254.0888 | 6.4388 |
| MT00307 | cAMP | 92 | Purine nucleotides | Cyclic purine nucleotides | 1.0673 | 0.0005 | 979.9200 | 328.0444 | 9.9324 |
| MT03210 | Adenosine phosphosulfate | HMDB0001003 | Purine nucleotides | Purine ribonucleotides | 1.1346 | 0.0000 | 977.2900 | 426.0114 | 7.9864 |
| MT03240 | Lithocholic acid glycine conjugate | HMDB0000698 | Steroids and steroid derivatives | Bile acids, alcohols and derivatives | 1.0952 | 0.0002 | 738.8700 | 478.3159 | 1.7332 |
| MT00537 | SM(d18:0/20:4(6E,8Z,11Z,13E)-2OH(5S,15S)) | HMDB0290488 | Unclassified | Unclassified | -1.3303 | 0.0000 | 841.0700 | 784.5732 | 0.9640 |
| MT02270 | PG(18:1(9Z)-O(12,13)/18:0) | HMDB0268822 | Unclassified | Unclassified | 1.2103 | 0.0001 | 878.0800 | 835.5315 | 1.0819 |
| MT02926 | PC(TXB2/18:1(9Z)) | HMDB0286454 | Unclassified | Unclassified | 1.0085 | 0.0025 | 946.6900 | 891.6064 | 5.8305 |

**Table S12. Detailed information and statistical summary of differential metabolites between the Gentiobiose fermentation group and the blank control.**

| **No.** | **Metabolites** | **Compound ID** | **Class** | **Sub Class** | **logFC** | **adj. *p*-value** | **Hotelling-T2** | **m/z** | **Retention time (min)** |
| --- | --- | --- | --- | --- | --- | --- | --- | --- | --- |
| MT00004 | L-Proline | 29 | Carboxylic acids and derivatives | Amino acids, peptides, and analogues | 1.4217 | 0.0000 | 1534.6000 | 116.0704 | 6.3958 |
| MT00044 | Saccharopine | 383 | Carboxylic acids and derivatives | Amino acids, peptides, and analogues | 1.2150 | 0.0006 | 1507.6000 | 275.1243 | 9.6176 |
| MT00125 | Thiazolidine-4-carboxylic acid | HMDB0258979 | Carboxylic acids and derivatives | Amino acids, peptides, and analogues | 1.4600 | 0.0000 | 845.6000 | 134.0264 | 4.0300 |
| MT00138 | 2-Hydroxy-L-methionine | HMDB0246915 | Carboxylic acids and derivatives | Amino acids, peptides, and analogues | 1.4918 | 0.0000 | 2005.0000 | 148.0420 | 2.1777 |
| MT00142 | Hydroxyprolylhydroxyproline | HMDB0028864 | Carboxylic acids and derivatives | Amino acids, peptides, and analogues | 1.2855 | 0.0000 | 1095.7000 | 225.0871 | 1.8920 |
| MT00186 | S-Prenyl-L-cysteine | HMDB0012286 | Carboxylic acids and derivatives | Amino acids, peptides, and analogues | 1.5009 | 0.0000 | 1994.3000 | 190.0887 | 1.5974 |
| MT00193 | Asparaginyl-Proline | HMDB0028739 | Carboxylic acids and derivatives | Amino acids, peptides, and analogues | 1.1155 | 0.0026 | 1406.6000 | 210.0873 | 6.0150 |
| MT00226 | Serylhydroxyproline | HMDB0029040 | Carboxylic acids and derivatives | Amino acids, peptides, and analogues | 1.1328 | 0.0009 | 1201.5000 | 217.0820 | 5.8876 |
| MT00251 | Thr-Leu | HMDB0259044 | Carboxylic acids and derivatives | Amino acids, peptides, and analogues | 1.1467 | 0.0007 | 889.7100 | 231.1341 | 5.8706 |
| MT00391 | L-Cystine | HMDB0000192 | Carboxylic acids and derivatives | Amino acids, peptides, and analogues | 1.1824 | 0.0002 | 935.4000 | 239.0157 | 9.7864 |
| MT00554 | Methyl glycinate | HMDB0254591 | Carboxylic acids and derivatives | Amino acids, peptides, and analogues | 1.5193 | 0.0000 | 1286.2000 | 134.0457 | 3.4212 |
| MT00595 | Methionyl-Threonine | HMDB0028983 | Carboxylic acids and derivatives | Amino acids, peptides, and analogues | 1.1351 | 0.0011 | 936.0700 | 249.0908 | 5.8706 |
| MT00679 | Histidylarginine | HMDB0028879 | Carboxylic acids and derivatives | Amino acids, peptides, and analogues | 1.0711 | 0.0003 | 998.9600 | 334.1594 | 5.9332 |
| MT00982 | N-(Mercaptoacetyl)glycine | HMDB0255024 | Carboxylic acids and derivatives | Amino acids, peptides, and analogues | 1.0752 | 0.0010 | 852.7200 | 297.0213 | 6.7367 |
| MT02330 | Asparaginyl-Serine | HMDB0028740 | Carboxylic acids and derivatives | Amino acids, peptides, and analogues | 1.0197 | 0.0005 | 909.2900 | 218.0773 | 6.0539 |
| MT02449 | N-Acetyl-3-(nitrosulfanyl)-L-valine | HMDB0244752 | Carboxylic acids and derivatives | Amino acids, peptides, and analogues | 1.0894 | 0.0008 | 1041.3000 | 235.0386 | 7.3715 |
| MT02453 | Histidylserine | HMDB0028894 | Carboxylic acids and derivatives | Amino acids, peptides, and analogues | 1.2350 | 0.0000 | 925.9800 | 243.1080 | 7.6707 |
| MT02454 | Asparaginyl-Threonine | HMDB0028741 | Carboxylic acids and derivatives | Amino acids, peptides, and analogues | 1.4447 | 0.0000 | 1909.6000 | 251.1359 | 7.8763 |
| MT02530 | Ethiin | HMDB0037057 | Carboxylic acids and derivatives | Amino acids, peptides, and analogues | 1.4778 | 0.0000 | 1972.2000 | 148.0420 | 3.3606 |
| MT02547 | Threoninyl-Aspartate | HMDB0304789 | Carboxylic acids and derivatives | Amino acids, peptides, and analogues | 1.4428 | 0.0000 | 1782.9000 | 235.0914 | 5.9161 |
| MT02679 | Threonylhistidine | HMDB0029063 | Carboxylic acids and derivatives | Amino acids, peptides, and analogues | 1.1510 | 0.0002 | 862.8500 | 257.1235 | 7.1183 |
| MT01333 | Methylmalonic acid | 3712 | Carboxylic acids and derivatives | Dicarboxylic acids and derivatives | 1.5142 | 0.0000 | 1227.5000 | 136.0610 | 3.4292 |
| MT00828 | Sodium citrate | HMDB0303752 | Carboxylic acids and derivatives | Tricarboxylic acids and derivatives | -1.5241 | 0.0000 | 2104.3000 | 259.9884 | 1.3380 |
| MT00835 | Indolo[2,1-b]quinazoline-6,12-dione | HMDB0253473 | Diazanaphthalenes | Benzodiazines | 1.8389 | 0.0000 | 2006.0000 | 495.1107 | 11.7079 |
| MT01910 | 4,5-Dihydro-2,5-dimethyl-4-oxo-3-furanyl butyrate | HMDB0303217 | Fatty Acyls | Fatty acid esters | 1.1198 | 0.0002 | 1086.8000 | 237.0526 | 7.5155 |
| MT00852 | Succinamic acid | HMDB0258538 | Fatty Acyls | Fatty acids and conjugates | 1.1210 | 0.0004 | 1165.2000 | 233.0770 | 5.9046 |
| MT03069 | PC(20:4(8Z,11Z,14Z,17Z)/0:0) | LMGP01050140 | Glycerophospholipids | Glycerophosphocholines | 1.3746 | 0.0000 | 1786.9000 | 582.2980 | 1.2897 |
| MT02808 | LysoPE(0:0/15:0) | HMDB0011472 | Glycerophospholipids | Glycerophosphoethanolamines | 1.4306 | 0.0000 | 1945.5000 | 438.2647 | 1.3380 |
| MT02139 | PGP(18:3(9Z,12Z,15Z)/22:6(4Z,7Z,10Z,13Z,16Z,19Z)) | HMDB0013591 | Glycerophospholipids | Glycerophosphoglycerophosphates | 1.1003 | 0.0001 | 925.5500 | 877.4403 | 7.3291 |
| MT00638 | 2-Carboxyoxypropanoic acid | HMDB0257720 | Organic carbonic acids and derivatives | Carbonic acid monoesters | 1.4455 | 0.0000 | 1653.1000 | 152.0558 | 2.2974 |
| MT00555 | Ciliatine | HMDB0011747 | Organic phosphonic acids and derivatives | Organic phosphonic acids | 1.5667 | 0.0000 | 1549.3000 | 170.0225 | 3.4212 |
| MT00430 | Glycerophosphoglycerol | HMDB0240316 | Organic phosphoric acids and derivatives | Phosphate esters | 1.4061 | 0.0000 | 1055.3000 | 245.0423 | 10.4996 |
| MT02984 | o-Cresol sulfate | HMDB0240655 | Organic sulfuric acids and derivatives | Arylsulfates | 1.4862 | 0.0000 | 2027.4000 | 206.0472 | 1.7338 |
| MT02332 | 6-amino-5[N-methylformylamino]-1-methyluracil | HMDB0059771 | Organonitrogen compounds | N-arylamides | 1.4916 | 0.0000 | 1973.0000 | 199.0817 | 6.0702 |
| MT00208 | D-Glucamine | HMDB0246693 | Organooxygen compounds | Carbohydrates and carbohydrate conjugates | 1.1161 | 0.0003 | 869.3300 | 164.0921 | 1.6316 |
| MT00332 | Lacdinac | HMDB0253938 | Organooxygen compounds | Carbohydrates and carbohydrate conjugates | 1.4029 | 0.0000 | 1221.3000 | 469.1658 | 6.4388 |
| MT00450 | 2,3,4-Trihydroxybutanoic acid | HMDB0245425 | Organooxygen compounds | Carbohydrates and carbohydrate conjugates | 1.4123 | 0.0000 | 1281.4000 | 135.0297 | 2.0977 |
| MT02198 | 5-phospho-alpha-D-ribose 1-diphosphate | HMDB0304220 | Organooxygen compounds | Carbohydrates and carbohydrate conjugates | 1.5196 | 0.0000 | 2085.4000 | 383.9035 | 3.9060 |
| MT01195 | 6-hydroxy-2-cyclohexen-one-carboxylate | HMDB0304226 | Organooxygen compounds | Carbonyl compounds | 1.0207 | 0.0017 | 882.8400 | 156.0413 | 6.6187 |
| MT00313 | Carnosine | HMDB0000033 | Peptidomimetics | Hybrid peptides | 1.0851 | 0.0004 | 1267.4000 | 225.0984 | 7.4565 |
| MT02412 | 7,8-dihydro-L-Biopterin | 5106 | Pteridines and derivatives | Pterins and derivatives | 1.1648 | 0.0001 | 1037.5000 | 284.0993 | 4.5153 |
| MT03316 | xanthurenic acid 8-O-sulfate | HMDB0059741 | Quinolines and derivatives | Quinoline carboxylic acids | 1.3876 | 0.0000 | 880.2300 | 284.9936 | 9.9888 |
| MT01943 | 3alpha,12beta-Dihydroxy-5beta-chol-6-en-24-oic Acid | LMST04010221 | Steroids and steroid derivatives | Bile acids and derivatives | -1.4935 | 0.0000 | 1212.9000 | 389.2685 | 0.9014 |
| MT03372 | Cholylarginine | HMDB0242375 | Steroids and steroid derivatives | Bile acids, alcohols and derivatives | 1.4782 | 0.0000 | 1882.2000 | 565.3933 | 5.9846 |
| MT00008 | AMINOHYDROXYBUTYRIC ACID | 44197 | Unclassified | Unclassified | 1.2970 | 0.0000 | 1178.5000 | 120.0652 | 6.1043 |
| MT00014 | (R)-(+)-2-Pyrrolidone-5-carboxylic acid | 63632 | Unclassified | Unclassified | 1.3929 | 0.0000 | 1143.8000 | 130.0493 | 1.2555 |
| MT00137 | Cholylglutamic acid | HMDB0242384 | Unclassified | Unclassified | 1.3807 | 0.0000 | 847.3500 | 536.3212 | 1.2420 |
| MT00537 | SM(d18:0/20:4(6E,8Z,11Z,13E)-2OH(5S,15S)) | HMDB0290488 | Unclassified | Unclassified | -1.4159 | 0.0000 | 950.0400 | 784.5732 | 0.9640 |
| MT02926 | PC(TXB2/18:1(9Z)) | HMDB0286454 | Unclassified | Unclassified | 1.1700 | 0.0002 | 1465.9000 | 891.6064 | 5.8305 |

**Table S13. Composition and selection rationale of the substrate-specific synthetic microbial communities.**

| **Target SynCom** | **Member** | **gLV-nominated responder** | **Direct utilization of cognate disaccharide in monoculture** | **Basis for inclusion** | **Proposed role in the SynCom** |
| --- | --- | --- | --- | --- | --- |
| Isomaltose SynCom | *Bifidobacterium pseudocatenulatum* | Yes | Yes | Identified by Bayesian gLV modeling as an isomaltose-associated keystone responder; showed direct isomaltose utilization and increased GanO and oligo-1,6-glucosidase abundance | Early isomaltose responder; substrate uptake and α-1,6 glycosidic-bond cleavage; major acetate-associated member |
|  | *Megamonas funiformis* | Yes | Yes | Identified by Bayesian gLV modeling as an isomaltose-associated keystone responder; directly utilized isomaltose and showed a strong substrate-response coefficient | Rapid early substrate utilization and community expansion; contribution to broad SCFA production |
|  | *Ruminococcus torques* | Yes | Yes | Identified by Bayesian gLV modeling as an early isomaltose-associated keystone responder; directly utilized isomaltose and expressed ChvE and oligo-1,6-glucosidase-associated proteins | Early α-1,6 substrate recognition and utilization |
|  | *Roseburia intestinalis* | No | Yes | Included as a functionally informed cultivable proxy for the unresolved Lachnospiraceae-affiliated responder based on shared taxonomic affiliation, published α-1,6-linked carbohydrate utilization and butyric acid-producing capacity, and direct isomaltose utilization in monoculture. | Functional replacement for the family-level Lachnospiraceae responder; late-stage butyric acid-associated member |
| Cellobiose SynCom | *Faecalibacterium prausnitzii* | Yes | Yes | Identified by Bayesian gLV modeling as a cellobiose-associated keystone responder; directly utilized cellobiose and expressed cellobiose-related transport and cleavage proteins | Late-stage cellobiose-associated responder and major butyric acid-producing member |
|  | *Megamonas funiformis* | Yes | Yes | Identified by Bayesian gLV modeling as a cellobiose-associated keystone responder; directly utilized cellobiose and showed increased CelB abundance | Early substrate utilization and contribution to community growth and SCFA formation |
|  | *Blautia obeum* | Yes | Yes | Identified by Bayesian gLV modeling as an early cellobiose-associated keystone responder; directly utilized cellobiose and expressed CebE or ChvE-like proteins, cellobiose phosphorylase, and β-glucosidase | Early cellobiose uptake and cleavage; potential intermediate-producing member |
|  | *Faecalibacillus intestinalis* | Yes | Yes | Identified by Bayesian gLV modeling as an early cellobiose-associated keystone responder; directly utilized cellobiose and expressed ABC.MS.S, CelB, and β-glucosidase | Early substrate capture and β-1,4 glycosidic-bond cleavage |
| Gentiobiose SynCom | *Bifidobacterium pseudocatenulatum* | Yes | Yes | Identified by Bayesian gLV modeling as the dominant gentiobiose-associated keystone responder; directly utilized gentiobiose and expressed ABC transport and β-glucosidase-associated proteins | Primary β-1,6 disaccharide responder; sustained substrate utilization and acetate-associated output |
|  | *Faecalibacillus intestinalis* | Yes | Yes | Identified by Bayesian gLV modeling as an early gentiobiose-associated keystone responder; directly utilized gentiobiose and expressed ABC transporter and β-glucosidase-associated proteins | Early substrate utilization and β-1,6 glycosidic-bond cleavage |
|  | *Bacteroides thetaiotaomicron* | No | Not tested in monoculture | Included as a putative functional complement based on published extracellular hydrolytic capacity toward β-1,6-linked glucans and the gentiobiose-associated increase in SusC-like, SusD-like, and β-1,6-related glycan-processing proteins assigned to this species; direct gentiobiose utilization was not tested. | Auxiliary extracellular substrate recognition and β-1,6 glycan-processing capacity |
|  | *Faecalibacterium prausnitzii* | No | No, not tested with gentiobiose | Included as a metabolic complement because of its established butyric acid-producing capacity and its late-stage association with butyric acid output | Downstream butyric acid-associated member and metabolic complement to acetate-producing responders |

**Table S14. Information on bacterial strains used in this study.**

| **Microbial strain** | **Strain number** | **Pre-culture medium** |
| --- | --- | --- |
| *Holdemanella biformis* | DSM 3989 | CMC |
| *Bifidobacterium pseudocatenulatum* | DSM 20438 | CMC |
| *Megamonas funiformis* | DSM 19343 | CMC |
| *Faecalibacterium prausnitzii* | DSM 107838 | CMC |
| *Ruminococcus torques* | ATCC 27756 | CMC |
| *Bacteroides thetaiotaomicron* | VPI 5482 | CMC |
| *Blautia obeum* | DSM 25238 | CMC |
| *Faecalibacillus intestinalis* | KCTC 15631 | CMC |
| *Roseburia intestinalis* | DSM 14610 | RCM |

**Table S15. Primer sequences used for (A) bacterial absolute quantification sequencing, (B) qPCR-based absolute quantification and standard construction, and (C) mouse gene expression analysis.**

**Primer sequences used for (A) bacterial absolute quantification sequencing**

| **Target gene** | **Forward primer (5'–3')** | **Reverse primer (5'–3')** |
| --- | --- | --- |
| 16S rRNA | AGRGTTTGATYNTGGCTCAG | TASGGHTACCTTGTTASGACTT |

**Primer sequences used for (B) qPCR-based absolute quantification and standard construction**

| **Target strain** | **Forward primer (5'–3')** | **Reverse primer (5'–3')** | **Target Gene (16S rRNA: Y/N)** | **Amplicon Size (bp)** | **Gene Copy**  **Number per**  **Genome** |
| --- | --- | --- | --- | --- | --- |
| *B. pseudocatenulatum* | CGGATGCTCCGACTCCT | CGAAGGCTTGCTCCCGAT | Y | 286 | 6 |
| *F. prausnitzii* | TGTAAACTCCTGTTGTTGAGGAAGATAA | GCGCTCCCTTTACACCCA | Y | 131 | 6 |
| *R. intestinalis* | GCGGTRCGGCAAGTCTGA | CCTCCGACACTCTAGTMCGAC | Y | 82 | 6 |
| *R. torques* | CCGTTCCTGACAACATTAG | GCCGATTCTCTTTCCTATATC | N | 145 | 1 |
| *B. thetaiotaomicron* | TCGCCTTTTTCAGATCAGTAGTTGG | ACGAAAATGGAGTTGAATGGAATAAGTT | N | 165 | 1 |
| *F. intestinalis* | TATTGGGCGTAAAGAGG | GCGTGGACTACTAGGGTAT | Y | 248 | 9 |
| *M. funiformis* | TATTGGGCGTAAAGGGAGCG | AAACCAGTTCGGACCCCATC | Y | 87 | 6 |
| *B. obeum* | TGGGTGTAAAGGGAGCGTAG | CTTTCGAGCCTCAACGTCAG | Y | 205 | 6 |

**Primer sequences used for (C) mouse gene expression analysis**

| **Target gene** | **Forward primer (5'–3')** | **Reverse primer (5'–3')** |
| --- | --- | --- |
| *ZO-1* | GCGGAGCCATGGATTGCAC | CTCTTCCTTGATACCAGGCCC |
| *Occludin* | TGAGCGACTGGTGGGAGGAG | GCAAGAGAGGCATCCTCACC |
| *Claudin-1* | GTTTGCAGAGACCCCATCAC | AGAAGCCAGGATGAAACCCA |
| *Claudin-2* | CAACTGGTGGGCTACATCCTA | CCCTTGGAAAAGCCAACCG |
| *β-actin* | AACACCCCAGCCATGTACG | ATGTCACGCACGATTTCCC |
